# Supplementary material for: Hidden threats in urban environments: Angiostrongylus cantonensis in Banda Aceh’s cityscape
Source: PLoS Negl Trop Dis. 2025 Oct 30;19(10):e0013634. doi: 10.1371/journal.pntd.0013634 (PMC12588454; doi:10.1371/journal.pntd.0013634)

PLOS Neglected Tropical Diseases

Hidden Threats in Urban Environments: Angiostrongylus cantonensis in Banda Aceh's Cityscape

--Manuscript Draft--

Manuscript Number:	PNTD-D-25-00434
Full Title:	Hidden Threats in Urban Environments: Angiostrongylus cantonensis in Banda Aceh's Cityscape
Short Title:	Hidden Threats in Urban Environments: Angiostrongylus cantonensis in Banda Aceh
Article Type:	Research Article
Keywords:	Angiostromgylus cantonensis, Rat Lungworm, Public Health, Snails, Food-borne Nematode
Abstract:	Angiostrongylus cantonensis is a mollusk-borne parasitic nematode originating in Southeast Asia. Commonly known as the rat lungworm, it uses rats as definitive hosts, though other mammals, including humans, can be infected and typically suffer from neurological disorders. This study focuses on the parasite's presence in its gastropod intermediate hosts in several urban and rural areas in Aceh province, Sumatra, Indonesia. Samples of Lissachatina fulica (161) and Pomacea sp. (90) were collected in eight localities in Banda Aceh. Additionally, 531 edible freshwater snails belonging to the genus Sulcospira sp. from three different localities in Aceh province were obtained in wet markets. All samples were examined by LAMP and qPCR for the A. cantonensis DNA. No samples of Sulcospira sp. and Pomacea sp. tested positive. 13.4% of L. fulica tested positive, with the highest prevalence in urban areas of Banda Aceh. The ITS1 sequences obtained from positive samples using conventional PCR confirmed 100% identity with A. cantonensis. The present study confirms, for the first time, the presence of the zoonotic parasite A. cantonensis in Banda Aceh, Sumatra, Indonesia. Notably, the handling and consumption of snails sold at wet markets do not appear to increase the risk of eosinophilic meningitis in this region. However, the relatively high prevalence of A. cantonensis in urban land snails underscores the need for continued vigilance and public health awareness.
Additional Information:	
Question	Response
**Government Employee**<br><br>Are you or any of the contributing authors an employee of the United States government?<br><br>Manuscripts authored by one or more US Government employees are not copyrighted, but are licensed under a CC0 Public Domain Dedication, which allows unlimited distribution and reuse of the article for any lawful purpose. This is a legal requirement for US Government employees.<br><br>This will be typeset if the manuscript is accepted for publication.	No - No authors are employees of the U.S. government.

Powered by Editorial Manager® and ProduXion Manager® from Aries Systems Corporation

**Financial Disclosure**<br><br>Enter a financial disclosure statement that describes the sources of funding for the work included in this submission. Review the submission guidelines for detailed requirements. View published research articles from *PLOS Neglected Tropical Diseases* for specific examples.<br><br>This statement is required for submission and **will appear in the published article** if the submission is accepted. Please make sure it is accurate.<br><br>**Funded studies**<br>Enter a statement with the following details:<br>• Initials of the authors who received each award<br>• Grant numbers awarded to each author<br>• The full name of each funder<br>• URL of each funder website<br>• Did the sponsors or funders play any role in the study design, data collection and analysis, decision to publish, or preparation of the manuscript?<br><br>Did you receive funding for this work?	Yes
Please add funding details.<br>as follow-up to "**Financial Disclosure**<br><br>Enter a financial disclosure statement that describes the sources of funding for the work included in this submission. Review the submission guidelines for detailed requirements. View published research articles from *PLOS Neglected Tropical Diseases* for specific examples.<br><br>This statement is required for submission and **will appear in the published article** if the submission is accepted. Please make sure it is accurate.	The study was supported by SEAEUROPEJFS19IN-053 and the Czech Science Foundation grant no. 22-26136S. Work of Lucia Anettova and Anna Sipkova was also supported by Specific research – support of student projects, no. MUNI/A/1602/2023.

Powered by Editorial Manager® and ProduXion Manager® from Aries Systems Corporation

**Funded studies**<br>Enter a statement with the following details:<br>• Initials of the authors who received each award<br>• Grant numbers awarded to each author<br>• The full name of each funder<br>• URL of each funder website<br>• Did the sponsors or funders play any role in the study design, data collection and analysis, decision to publish, or preparation of the manuscript?<br><br>Did you receive funding for this work?"	
Please select the country of your main research funder (please select carefully as in some cases this is used in fee calculation).<br>as follow-up to "**Financial Disclosure**<br><br>Enter a financial disclosure statement that describes the sources of funding for the work included in this submission. Review the submission guidelines for detailed requirements. View published research articles from *PLOS Neglected Tropical Diseases* for specific examples.<br><br>This statement is required for submission and **will appear in the published article** if the submission is accepted. Please make sure it is accurate.<br><br>**Funded studies**<br>Enter a statement with the following details:<br>• Initials of the authors who received each award<br>• Grant numbers awarded to each author<br>• The full name of each funder<br>• URL of each funder website<br>• Did the sponsors or funders play any role in the study design, data collection and analysis, decision to publish, or preparation of the manuscript?<br><br>Did you receive funding for this work?"	CZECH REPUBLIC - CZ
**Competing Interests**	No competing interests.

Powered by Editorial Manager® and ProduXion Manager® from Aries Systems Corporation

On behalf of all authors, disclose any competing interests that could be perceived to bias this work.<br><br>This statement will be typeset if the manuscript is accepted for publication.<br><br>Review the instructions link below and PLOS NTDs' competing interests policy to determine what information must be disclosed at submission.	
**Data Availability**<br><br>Provide a **Data Availability Statement** in the box below. This statement should detail where the data used in this submission can be accessed. This statement will be typeset if the manuscript is accepted for publication.<br><br>Before publication, authors are required to make all data underlying their findings fully available, without restriction. Review our PLOS Data Policy page for detailed information on this policy. Instructions for writing your Data Availability statement can be accessed via the Instructions link below.	All data generated or analyzed during this study are included in this published article.

Main Document
Click here to access/download;Manuscript;Aceh_MS_PLoS_Angiostrongylus

Hidden Threats in Urban Environments: *Angiostrongylus cantonensis* in Banda Aceh's Cityscape

Lucia Anettová[1,2]◑*, Anna Šipková[2]◑, Vojtech Baláž[3], Muhammad Hambal[4], Radovan Coufal[2], Jana Kačmaříková[5], Henni Vanda[4], Wahyu Eka Sari[4], David Modrý[1,2,6]

1 Department of Veterinary Sciences/Faculty of Agrobiology, Food and Natural Resources, Czech University of Life Sciences, Prague, Czech Republic
2 Department of Botany and Zoology/Faculty of Science, Masaryk University, Brno, Czech Republic
3 Department of Ecology and Diseases of Zoo Animals, Game, Fish and Bees, Faculty of Veterinary Hygiene and Ecology, University of Veterinary Sciences Brno, Czech Republic
4 Faculty of Veterinary Medicine/University Syiah Kuala, Banda Aceh, Indonesia
5 Department of Pathology and Parasitology/Faculty of Veterinary Medicine, University of Veterinary Sciences Brno, Czech Republic
6 Institute of Parasitology/Biology Center/Czech Academy of Sciences, České Budějovice, Czech Republic

◑These authors contributed equally to this work.
* lucia.anettova@gmail.com

Abstract

Angiostrongylus cantonensis is a mollusk-borne parasitic nematode originating in Southeast Asia. Commonly known as the rat lungworm, it uses rats as definitive hosts, though other mammals, including humans, can be infected and typically suffer from neurological disorders. This study focuses on the parasite's presence in its gastropod intermediate hosts in several urban and rural areas in Aceh province, Sumatra, Indonesia. Samples of *Lissachatina fulica* (161) and *Pomacea* sp. (90) were collected in eight localities in Banda Aceh. Additionally, 531 edible freshwater snails belonging to the genus *Sulcospira* sp. from three different localities in Aceh province were obtained in wet markets. All samples were examined by LAMP and qPCR for the *A. cantonensis* DNA. No samples of *Sulcospira* sp. and *Pomacea* sp. tested positive. 13.4% of *L. fulica* tested positive, with the highest prevalence in urban areas of Banda Aceh. The ITS1 sequences obtained from positive samples using conventional PCR confirmed 100% identity with *A. cantonensis*. The present study confirms, for the first time, the presence of the zoonotic parasite *A. cantonensis* in Banda Aceh, Sumatra, Indonesia. Notably, the handling and consumption of snails sold at wet markets do not appear to increase the risk of eosinophilic meningitis in this region. However, the relatively high prevalence of *A. cantonensis* in urban land snails underscores the need for continued vigilance and public health awareness.

Introduction

The rat lungworm *Angiostrongylus cantonensis* is a food-borne zoonotic metastrongyloid nematode first described in Canton, China (1). However, it has expanded throughout the world and is considered an emerging pathogen (2), causing

eosinophilic meningitis in humans and similar neurological disorders in numerous mammalian and avian species considered aberrant hosts (3; 4; 5; 6; 7).

Cases of human eosinophilic meningitis likely caused by *A. cantonensis* have been reported from Southeast Asia since the 1960s (8; 9). The parasite's presence in both, definitive and intermediate hosts or in humans (as its accidental hosts) have been confirmed on most of the main islands of Indonesia (9; 10; 11; 12). In Sumatra, eight human cases of eosinophilic meningitis likely caused by *A. cantonensis*, were first reported in Kisaran, North Sumatra Province (13). The parasite was also identified in rats *Rattus tiomanicus jalorensis* and *Rattus rattus diardii*, originating from the surroundings of Medan, North Sumatra Province (14). Although no further human infections have been documented, the parasite has been detected in rats and snails (*Lissachatina fulica*) across the island, particularly in South Sumatra Province (Lubuk Linggau, Baturaja, and Martapura) and Lampung Province (10; 15; 16). However, the northwestern part of Sumatra remains largely unstudied.

The giant terrestrial African snail (*L. fulica*, Fig. 1) and the freshwater apple snail (*Pomacea* spp., Fig. 2) are among notable invasive mollusks associated with the zoonotic transmission of *A. cantonensis* to humans (2; 17; 18). Resulting from a successful invasion, the abundance of these gastropods in Aceh province is high and these species could serve as suitable terrestrial and aquatic intermediate hosts for *A. cantonensis* in the area. Although widely distributed in Aceh province, these mollusks are generally not used for human consumption, in contrast to freshwater *Sulcospira* spp. (Fig. 3) commonly consumed by the local population. However, the potential role of this species as an intermediate host for *A. cantonensis* remains undetermined.

Rats of the genus *Rattus* are definitive hosts for *Angiostrongylus cantonensis*, with gastropods serving as intermediate hosts. The parasite's life cycle involves first-stage larvae (L1) excreted in rat feces, developing into infective third-stage larvae (L3) within gastropods (19). Poikilothermic animals (including reptiles and amphibians) can act as paratenic hosts (20; 21; 22; 23), transmitting infection to humans and other mammals. Due to the invasion and spread of the parasite, facilitated by rats and gastropods, resulting disease is becoming increasingly common in humans and is considered one of the emerging infectious diseases (2).

Apart from direct ingestion of intermediate or paratenic hosts, food, water or hands contaminated with free L3 released from gastropods represent another route of transmission to humans (19; 24). In the human body, the parasites reach the brain in the same way as in rats and develop to the subadult stage. The immune reaction and the presence of larvae in the brain result in severe neurological symptoms, occasionally with fatal consequences (25).

Aceh Province is the northernmost part of Sumatra, characterized by a warm and humid climate that is conducive to the life cycle of *A. cantonensis*. Culinary habits in Aceh include the consumption of *Sulcospira* snails as well as other freshwater invertebrates and vertebrates, which are potential paratenic hosts for *A. cantonensis*, underscoring the need for ongoing monitoring and public health vigilance. Our study investigates the presence of *A. cantonensis* in intermediate hosts in urban, suburban and rural areas of Banda Aceh and potential risk for zoonotic infections. To the authors´ knowledge, this is the first study to investigate the presence of *A. cantonensis* in Aceh province.

Materials and methods

Sample collection

Landsnails *L. fulica* were collected in five localities, two urban: city center (1) and campus of the University Syiah Kuala (2); one suburban: Lambirah (3); and two rural: pastures near Kuede (4) and gardens by Nurul Qualbi (5). The urban locality in the city center was further divided into two sublocalities: park (1a) and riverside (1b) (Table 1, Fig. 4). The localities were characterized as urban, suburban or rural based on land use and infrastructure in the particular areas, taking into account population density in the areas. In each locality/sublocality, at least 30 snails were collected, except for the locality 4, where we were able to find only 11 individuals. All collected *L. fulica* were euthanized by freezing. The samples were first processed using the LAMP assay in pools. Tissue samples from each individual were also stored for further qPCR analysis if the pooled LAMP analysis was positive. All the *L. fulica* samples were examined in pools of three snails, pooling approximately 10 µg of a foot tissue from each snail.

Pomacea sp. were collected in three localities: two suburban (Lambirah and along Jl. Tgk. Bakurma) and one urban (University Syiah Kuala campus). Thirty snails were collected from each locality. All the aquatic snails were put into hard plastic zip bags in pools of three, filled with water up to one third of volume of the zip bag (approx. 50 mL). Shells of snails were crushed and one additional hour was provided for snail tissues to macerate and larvae of *A. cantonensis* to emerge. After an hour, 0.5 ml of sediment was taken from each bag for the DNA extraction for LAMP assay with PrepMan.

In wet markets, edible freshwater snails *Sulcospira* sp. (Fig. 3) from three different localities in Aceh province were obtained. 150 snails were obtained from Lhoong, Aceh Besar District, 91 from Leupung Subdistrict, Aceh Besar District and 290 from Lamno, Aceh Jaya District. The snails from wet markets were treated the same way as *L. fulica*, examined in pools of 10 individuals.

Molecular analysis and sequence data

DNA was extracted from snail tissues using PrepMan in a ratio of the tissue and the PrepMan reagent 1:1 in 2 mL vials, i.e., 50-150 L of PrepMan was used according to the tissue amount. The mixture for the DNA extraction was exposed to 99C in a water bath for 10 minutes and used immediately. The LAMP assays were performed using Genie® II, OptiGene, UK in 20 L reaction, as described by Baláž et al. (26).

DNA from pooled samples which tested positive with LAMP was subsequently extracted from individuals in the given pool using DNEasy Blood&Tissue (Qiagen, Germany) extraction kit with modification optimized for L3 larvae of *A. cantonensis*, when the pre-lyse phase was extended overnight. The qPCR assay was performed on BIO-RAD CFX96 Real Time system in a 20 L reaction, according to Sears et al. (27) 2021 and modified as in Anettová et al. (28) 2024. As positive controls, DNA from a single L3 larva of *A. cantonensis* extracted by the same method as samples and diluted 100x was used.

To confirm the identity of larvae as *A. cantonensis*, nested PCR was performed to obtain ITS1 sequences. This consisted of using specific primers targeting the ITS-1 region of *A. cantonensis* (29). PCR products were purified with ExoSAP-IT (Affymetrix, ThermoFisher Scientific, Czech Republic) and sequenced commercially at Macrogen Europe (Amsterdam, Netherlands) using the amplification primers. Sequences were assessed and trimmed manually in Geneious Prime 2024.0.5 (http://www.geneious.com) and checked against BLAST (30).

Additionally, SYBR Green-based quantitative real-time PCR (31) was performed on the DNA samples tested positive previously, to investigate possible co-occurence of *A.*

malaysiensis. The assay was run in duplicates and two sets of primers for the two nematodes were used. In case of confirmation of *A. cantonensis* the primers AC4_cytb_F (5AAT GTT TGT TGA GGC AGA TC 3) and AC5_cytb_R (5 GCT ACA ACA CCC ATA ACC T 3) were used as originally published. In case of *A. malaysiensis*, we have adjusted the length of primers, to cover more variable positions and increase annealing temperature, AM_cytb_Fnew (5´CGA GAT ATT TAT TGA GGC TGA TCC TTT GAC 3´) and AM_cytb_Rnew (5´GAC AAA ACC CTC ATC AAT AAA GCC A3´).

1 Results

The terrestrial *L. fulica* was the only species that tested positive for *A. cantonensis* DNA. Of the 164 specimens collected in Banda Aceh municipality and its surroundings, 22 (13.4%) were positive. The highest prevalence was observed in the city center sublocalities: 6 of 32 samples (18.8%; sublocality 1a) and 11 of 31 samples (35.5%; sublocality 1b). At campus of University Syiah Kuala, 3 of 30 samples (10%) were positive, with one additional sample yielding dubious result. Among rural localities, only Nurul Qualbi (Loc 5) showed positive results, with 2 of 30 samples (6. 7%) positive. No positive samples were found in Lambirah and Kuede. Aquatic snail *Sulcospira* sp. tested negative for *A. cantonensis* DNA in all examined localities. The results of *L. fulica* examination are summarized in Table 1; examples of positive and negative localities are shown in Fig. 3.

Five DNA sequences of length 322 to 554 bp from two different localities (City Center - park and Nurul Qualbi gardens) were obtained from *L. fulica* samples; BLAST analysis showed 100% identity with GenBank sequences of *A. cantonensis*. The sequences obtained were identical, and one representative sequence from each locality has been submitted to GenBank with accession numbers PV133028 and PV133029. All DNA samples analyzed using SYBR Green-based real-time PCR tested positive with primers specific for *A. cantonensis*, while none tested positive for *A. malaysiensis*.

Discussion

The study provides further data on distribution of *A. cantonensis* in Sumatra (13; 14), with first sequence data from this region. Our findings confirm the presence of *A. cantonensis* in the municipality of Banda Aceh, with DNA detected only in the terrestrial African giant snail *L. fulica* with a remarkable prevalence, especially in the city center. Conversely, all examined samples from aquatic gastropods tested negative, despite a relatively large sample size of edible aquatic snails, including *Sulcospira* sp. and *Pomacea* sp. These results suggest absence or a very low presence of *A. cantonensis* in the aquatic environment in tested localities of the Aceh province.

To confirm the taxonomic identity of the nematode and to exclude possible co-occurence with *A. malaysiensis* as *A. cantonensis*, ITS1 sequence were analyzed and the SYBR-Green based real-time PCR was conducted, providing an ultimate taxonomic identification. While the qPCR assay used is highly sensitive and specific (27), it is not suitable for distinguishing among the three sibling species: *A. cantonensis*, *A. malaysiensis* (which likely overlaps with *A. cantonensis* in Southeast Asia), and *A. mackerrasae* (found in Australia). Notably, both *A. cantonensis* and *A. malaysiensis* are zoonotic.

The absence of *A. cantonensis* in *Pomacea* sp. in areas where *L. fulica* tested positive was unexpected. This disparity may indicate low exposure of aquatic mollusks in tested localities to *A. cantonensis* larvae or that *Pomacea* sp. are less suitable intermediate hosts compared to *L. fulica*. Previous studies support the latter hypothesis,

Table 1. Sampling localities of *Lissachatina fulica* in Banda Aceh and its surroundings, with the categorization and numbers of samples tested positive by LAMP and qPCR.

Locality	Type	No. of Samples	No. Positive
(1a) City center (park)	urban	32	6
(1b) City center (riverside)	urban	31	11
(2) University Syiah Kuala	urban	30	3
(3) Lambirah	suburban	30	0
(4) Kuede	rural	11	0
(5) Nurul Qualbi gardens	rural	30	2

showing lower prevalence in *Pomacea* spp. compared to terrestrial gastropods such as *L. fulica* (32). However, no experimental studies have directly compared the suitability of these two intermediate hosts for supporting *A. cantonensis* larvae, highlighting a potential area for future research. Alternatively, this absence in aquatic hosts could also indicate an early stage of introduction in Aceh or environmental factors that limit the parasite's spread and development in these hosts and habitats. Further studies are needed to explore these factors and their influence on host-parasite dynamics.

Sulcospira sp., an edible aquatic snail frequently consumed as a traditional protein source in Aceh, tested negative for *A. cantonensis* DNA. To the authors' knowledge, this species has not previously been proven as an intermediate host. Despite a relatively large sample size, the examined material was limited to only three sources, all defined as extensive snail farms, which may not fully grasp the diversity of potential parasite-host associations. The rat lungworm is known for its low host specificity, utilizing a wide range of gastropod species as intermediate hosts (19) and the possibility of transmission through *Sulcospira* sp. should not be dismissed. Even when these snails are cooked, the risk of human infection during transportation and food preparation remains a concern, highlighting the need for further investigation into possible presence of *A. cantonensis* in the aquatic environment.

Our results emphasize predominant association of *A. cantonensis* with urban areas and its much lower prevalence in suburban and rural regions. This pattern may be driven by higher population densities of *L. fulica* in urban environments (33; 34), as this highly invasive species has a lower chance to be introduced directly into the natural or rural environment. However, as the snails and the parasite continue to spread, they are likely to eventually colonize these areas and find the environment suitable. Demonstrated presence in urban environment underscores risk of possible contaminative transmission.

Presence of *A. cantonensis* in this newly recognized area highlights the need for public health interventions, such as minimizing human contact with terrestrial snails, particularly in urban settings. Given the frequent consumption of freshwater gastropods and other aquatic hosts in Aceh, the potential for infection via aquatic pathways cannot be ignored. Further research should explore the prevalence of *A. cantonensis* in these hosts to assess the risk posed by local dietary practice (35; 36). Neuroangiostrongyliasis remains a significant public health concern and increased awareness is essential to mitigate its impact. Finally, the existence of underdiagnosed cases of eosinophilic meningitis in the region, particularly in areas with limited healthcare resources, should be considered (2; 37).

Acknowledgments

The authors thank Petr Janoš for creating the map of localities studied for the presence of *Angiostrongylus cantonensis* in *Lissachatina fulica* (Fig. 4).

Funding

The study was supported by SEAEUROPEJFS19IN-053 and the Czech Science Foundation grant no. 22-26136S. Work of Lucia Anettová and Anna Šipková was also supported by Specific research – support of student projects, no. MUNI/A/1602/2023.

Conflicts of Interest

The authors declare there are no conflicts of interest.

Figure Legend

Fig. 1 Invasive landsnail *Lissachatina fulica* found in the campus of the University Syiah Kuala, Banda Aceh, Indonesia.

Fig. 2 Invasive aquatic snail *Pomacea* sp. found in a pond of the rural area near Banda Aceh, Indonesia.

Fig. 3 Edible aquatic snail *Sulcospira* sp. from wet markets, originally from extensive snail farms in Aceh province, Indonesia.

Fig. 4 Sampling localities of *Lissachatina fulica* for the detection of *Angiostrongylus cantonensis* DNA in Banda Aceh, Indonesia.

References

1. Chen HT. Un nouveau nématode pulmonaire, *Pulmonema cantonensis*, ng, n. sp. Annales de Parasitologie humaine et comparée. 1935;13(4):312–317.

2. Cowie RH, Ansdell V, Dunavan CP, Rollins RL. Neuroangiostrongyliasis: global spread of an emerging tropical disease. The American Journal of Tropical Medicine and Hygiene. 2022;107(6):1166.

3. Costa LRR, McClure JJ, Snider TG, Stewart TB. Verminous meningoencephalomyelitis by *Angiostrongylus* (= *Parastrongylus*) *cantonensis* in an American Miniature Horse. Equine Veterinary Education. 2000;12(1):2–6. doi:10.1111/j.2042-3292.2000.tb01754.x.

4. Kim D, Stewart T, Bauer R, Mitchell M. *Parastrongylus* (= *Angiostrongylus*) *cantonensis* now endemic in Louisiana wildlife. Journal of Parasitology. 2002;88(5):1024–1026.

5. Monks DJ, Carlisle MS, Carrigan M, Rose K, Spratt D, Gallagher A, et al. *Angiostrongylus cantonensis* as a cause of cerebrospinal disease in a yellow-tailed black cockatoo (*Calyptorhynchus funereus*) and two tawny frogmouths (*Podargus strigoides*). Journal of Avian Medicine and Surgery. 2005;19(4):289–293.

6. Spratt DM. Species of *Angiostrongylus* (Nematoda: Metastrongyloidea) in wildlife: A review. International Journal for Parasitology: Parasites and Wildlife. 2015;4(2):178–189.

7. Gardiner CH, Wells S, Gutter AE, Fitzgerald L, Anderson DC, Harris RK, et al. Eosinophilic meningoencephalitis due to *Angiostrongylus cantonensis* as the cause of death in captive non-human primates. American Journal of Tropical Medicine and Hygiene. 1990;42(1):70–74. doi:10.4269/ajtmh.1990.42.70.

8. Jindrák K, Alicata JE. A case of parasitic eosinophilic meningo-encephalitis in Vietnam probably caused by *Angiostrongylus cantonensis*. Annals of Tropical Medicine and Parasitology. 1965;59:294–300.

9. Scrimgeour EM. Distribution of *Angiostrongylus cantonensis* in Papua New Guinea. Transactions of the Royal Society of Tropical Medicine and Hygiene. 1984;78(6):776–779. doi:10.1016/0035-9203(84)90017-8.

10. Carney WP, Stafford EE, Purnomo, Tanudjaja S. Angiostrongyliasis in Indonesia: additional geographic and host occurrence records. Southeast Asian Journal of Tropical Medicine and Public Health. 1978;9(4):516–519.

11. Uga S, Ono K, Kataoka N, Hasan H. Seroepidemiology of five major zoonotic parasite infections in inhabitants of Sidoarjo, East Java, Indonesia. Southeast Asian Journal of Tropical Medicine and Public Health. 1996;27(3):556–561.

12. Lim BL. Occurrence of *Angiostrongylus cantonensis* in rats around Kuching, Sarawak. Annals of Tropical Medicine Parasitology. 1967;61(4):429–431. doi:10.1080/00034983.1967.11686510.

13. Smit AM. Eosinophilic meningitis at Kisaran (Indonesia) and problem of its aetiology. Bulletin de la Société de Pathologie Exotique. 1962;55:720–722.

14. Kwo E, Kwo I. Occurrence of *Angiostrongylus cantonensis* in Rats in North Sumatra, Indonesia. The Journal of Parasitology. 1968;54(3):537–537. doi:10.2307/3277078.

15. Liat BL, et al. Studies of *Angiostrongylus cantonensis* and a *Malaysiensis* of Rodents Hosts in Two Transmigration Schemes in South Sumatera. Indonesian Bulletin of Health Research. 1978;6(1). doi:10.22435/bpk.v6i1Mar.342.

16. Stafford EE, Sukeri S, Sutanti T. The bandicoot rat, a new host record for *Angiostrongylus cantonensis* in Indonesia. Southeast Asian Journal of Tropical Medicine and Public Health. 1976;1:41–44.

17. de Brito FC, Joshi RC. The Golden Apple Snail *Pomacea canaliculata*: A review on invasion, dispersion and control. Outlooks on Pest Management. 2016;27(4):157–163.

18. Silva GM, Thiengo SC, Sierpe Jeraldo VL, Rego MIF, Silva ABP, Rodrigues PS, et al. The invasive giant African land snail, *Achatina fulica* (Gastropoda: Pulmonata): global geographical distribution of this species as host of nematodes of medical and veterinary importance. Journal of Helminthology. 2022;96:e86. doi:10.1017/S0022149X22000761.

19. Cowie RH. Pathways for transmission of angiostrongyliasis and the risk of disease associated with them. Hawaii Journal of Medicine Public Health. 2013;72(6 Suppl 2):70–74.

20. Anettová L, Izquierdo-Rodriguez E, Foronda P, Baláž V, Novotný L, Modrý D. Endemic lizard *Gallotia galloti* is a paratenic host of invasive *Angiostrongylus cantonensis* in Tenerife, Spain. Parasitology. 2022;149(7):934–939.

21. Ash LR. The occurrence of *Angiostrongylus cantonensis* in frogs of New Caledonia with observations on paratenic hosts of metastrongyles. The Journal of Parasitology. 1968; p. 432–436.

22. Rosen L, Chappell R, Laqueur GL, Wallace GD, Weinstein PP. Eosinophilic meningoencephalitis caused by a metastrongylid lung-worm of rats. Jama. 1962;179(8):620–624.

23. Turck HC, Fox MT, Cowie RH. Paratenic hosts of *Angiostrongylus cantonensis* and their relation to human neuroangiostrongyliasis globally. One Health. 2022; p. 100426.

24. Šipková A, Anettová L, Izquierdo-Rodriguez E, Velič V, Modrý D. Release of *Angiostrongylus cantonensis* larvae from live intermediate hosts under stress. Parasitology Research. 2024;123(5):1–7.

25. Kliks MM, Palumbo NE. Eosinophilic meningitis beyond the Pacific Basin: the global dispersal of a peridomestic zoonosis caused by *Angiostrongylus cantonensis*, the nematode lungworm of rats. Social Science Medicine. 1992;34(2):199–212. doi:10.1016/0277-9536(92)90097-a.

26. Baláž V, Rivory P, Hayward D, Jaensch S, Malik R, Lee R, et al. Angie-LAMP for diagnosis of human eosinophilic meningitis using dog as proxy: A LAMP assay for *Angiostrongylus cantonensis* DNA in cerebrospinal fluid. PLoS Neglected Tropical Diseases. 2023;17(5):e0011038.

27. Sears WJ, Qvarnstrom Y, Dahlstrom E, Snook K, Kaluna L, Baláž V, et al. AcanR3990 qPCR: a novel, highly sensitive, bioinformatically-informed assay to detect *Angiostrongylus cantonensis* infections. Clinical Infectious Diseases. 2021;73(7):e1594–e1600.

28. Anettová L, Kačmaříková J, Šipková A, Velič V, Cavallo L, Dyková I, et al. Low potential of fish as a source of infection with *Angiostrongylus cantonensis*. Food Quality and Safety. 2024; p. fyae042.

29. Izquierdo-Rodriguez E, Anettová L, Hrazdilová K, Foronda P, Modrý D. Range of metastrongylids (superfamily Metastrongyloidea) of public health and veterinary concern present in livers of the endemic lizard Gallotia galloti of Tenerife, Canary Islands, Spain. Parasites & Vectors. 2023;16(1):81.

30. Altschul SF, Gish W, Miller W, Myers EW, Lipman DJ. Basic local alignment search tool. Journal of molecular biology. 1990;215(3):403–410.

31. Jakkul W, Chaisiri K, Saralamba N, Limpanont Y, Dusitsittipon S, Charoennitiwat V, et al. Newly developed SYBR green-based quantitative real-time PCRs revealed coinfection evidence of *Angiostrongylus cantonensis* and *A. malaysiensis* in *Achatina fulica* existing in Bangkok metropolitan, Thailand. Food and waterborne parasitology. 2021;23:e00119.

32. Song L, Wang X, Yang Z, Lv Z, Wu Z. *Angiostrongylus cantonensis* in the vector snails *Pomacea canaliculata* and *Achatina fulica* in China: a meta-analysis. Parasitology Research. 2016;115:913–923.

33. Thiengo SC, Faraco FA, Salgado NC, Cowie RH, Fernandez MA. Rapid spread of an invasive snail in South America: the giant African snail, *Achatina fulica*, in Brasil. Biological Invasions. 2007;9:693–702.

34. Gamarra-Rueda R, García R, Restrepo-Rodas DC, Pérez-García J. First identification of *Angiostrongylus* spp. in *Lissachatina fulica* and *Cornu aspersum* in Antioquia, Colombia. Biomédica. 2024;44(3):416–424.

35. Ervilita R, Mardhiah A. Local Wisdom of the Aceh Jaya Society in terms of Indigenous Cuisine that can be learned in Chemistry Learning in Schools. In: Proceedings of International Conference on Multidiciplinary Research. vol. 5; 2022. p. 134–146.

36. Muchlisin ZA, Rinaldi F, Fadli N, Adlim M, Siti-Azizah MN. Food preference and diet overlap of two endemic and threatened freshwater fishes, depik (*Rasbora tawarensis*) and kawan (*Poropuntius tawarensis*) in Lake Laut Tawar, Indonesia. Aquaculture, Aquarium, Conservation & Legislation. 2015;8(1):40–49.

37. Pandian D, Najer T, Modrý D. An overview of *Angiostrongylus cantonensis* (Nematoda: Angiostrongylidae), an emerging cause of human angiostrongylosis on the Indian subcontinent. Pathogens. 2023;12(6):851.

Figure 1

Click here to access/download;Figure;Figure_1.tif

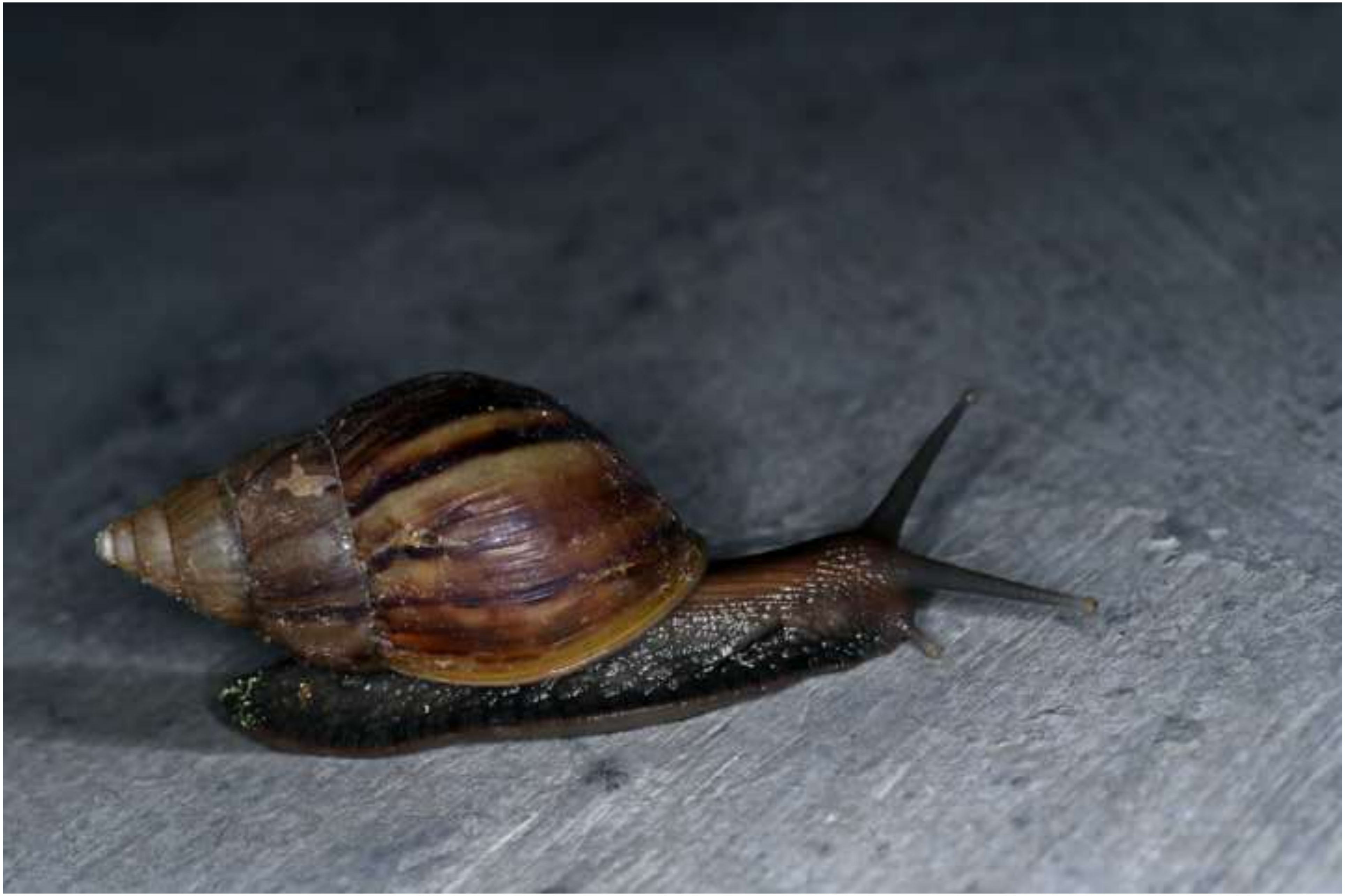

Figure 2

Click here to access/download;Figure;Figure_2.tif

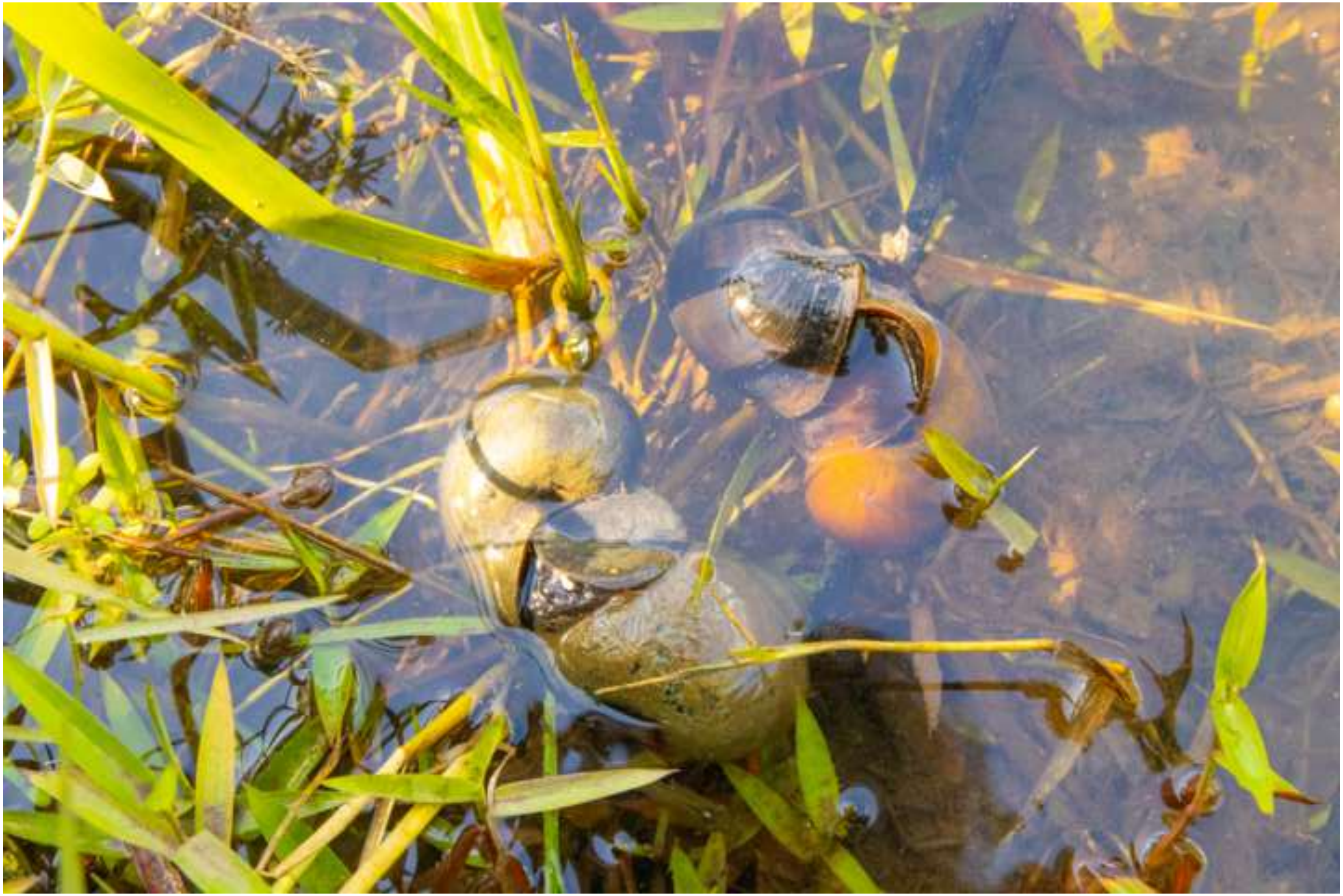

Figure 3

Click here to access/download;Figure;Figure_3.tif

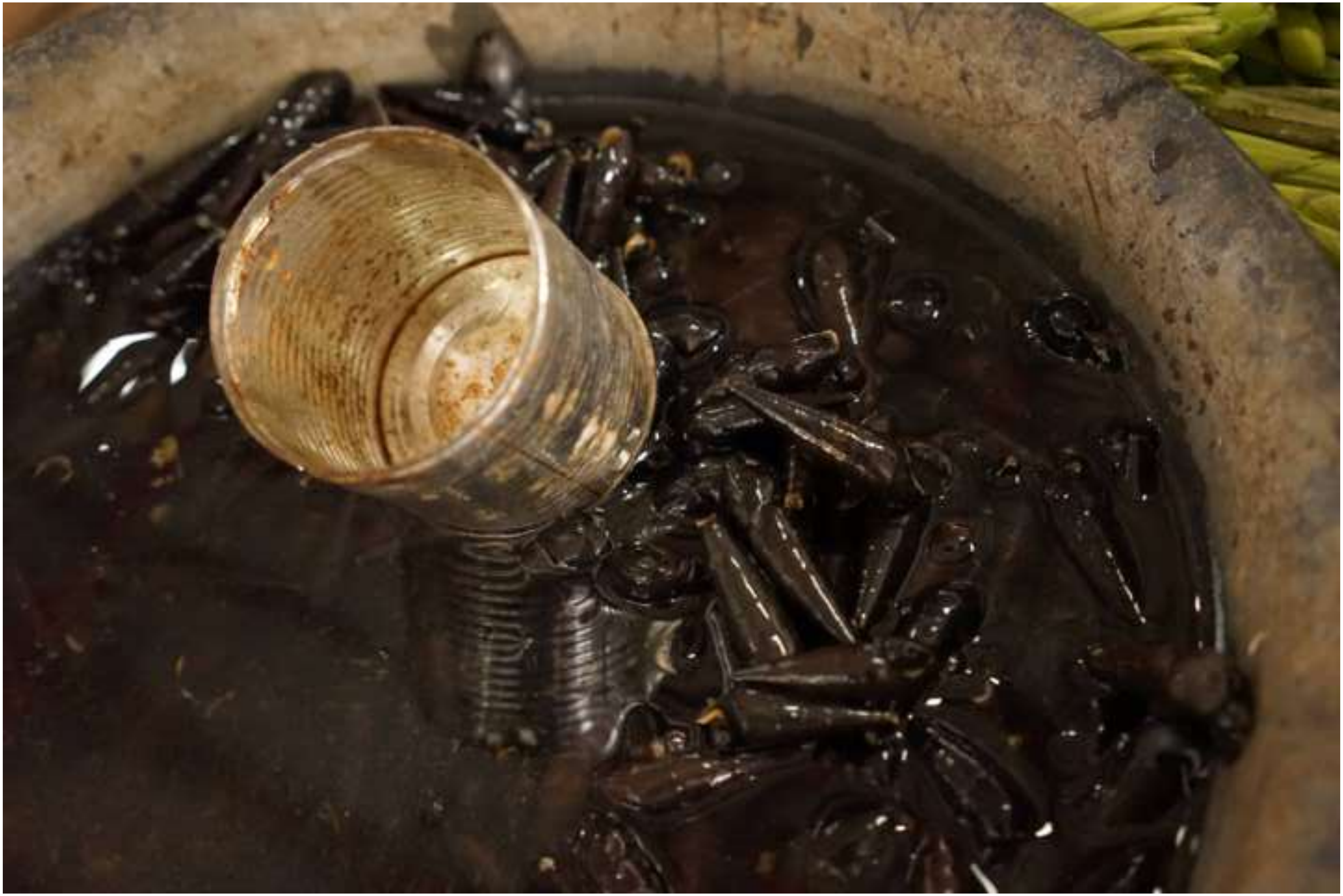

Figure 4

Click here to access/download;Figure;Figure_4.tif

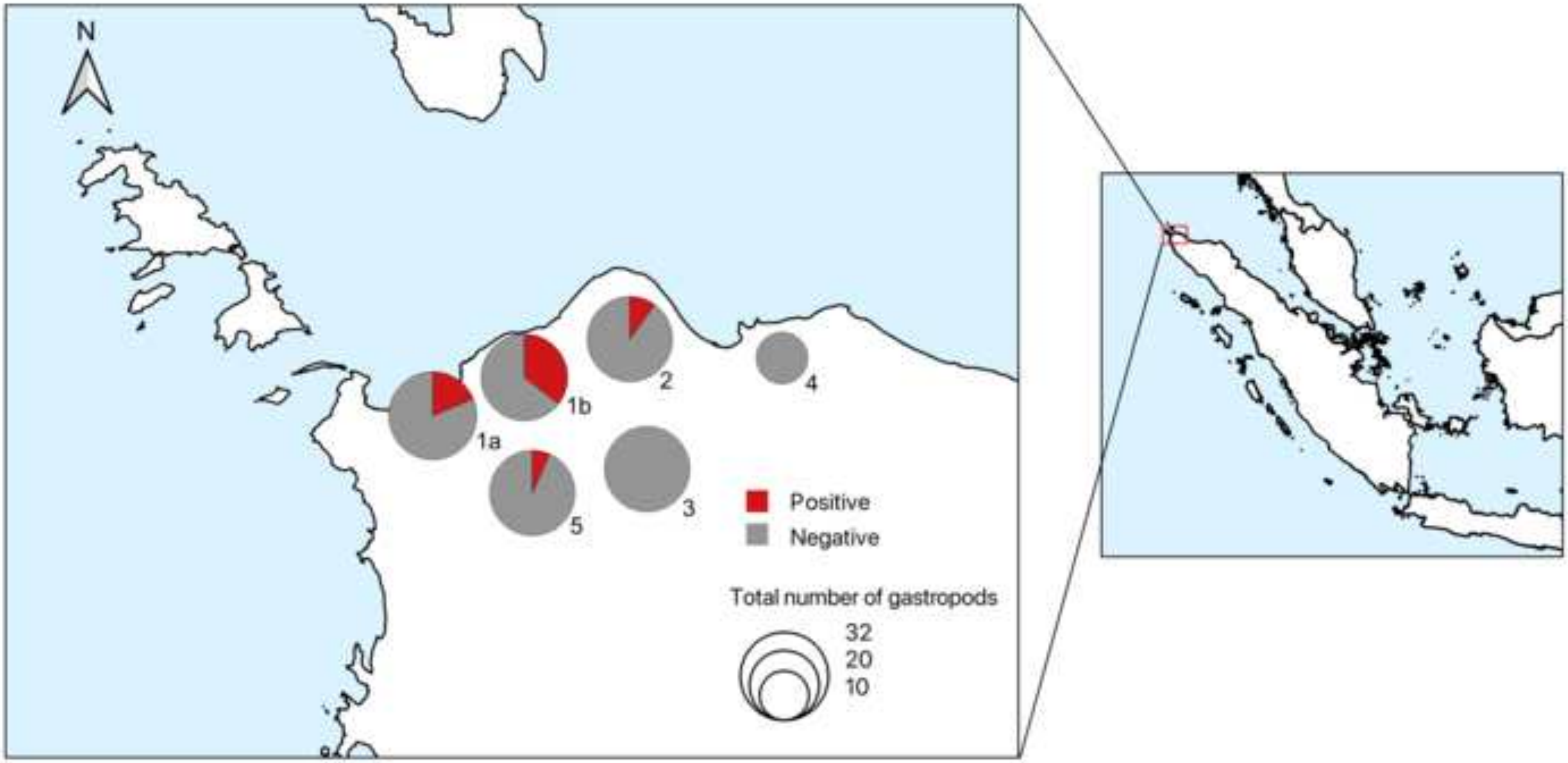

Supplement: Attachment — Submitted filename: PNTD-D-25-00434_reviewer.pdf [file pntd.0013634.s001.pdf]
