# Supplementary material for: Hidden threats in urban environments: Angiostrongylus cantonensis in Banda Aceh’s cityscape
Source: PLoS Negl Trop Dis. 2025 Oct 30;19(10):e0013634. doi: 10.1371/journal.pntd.0013634 (PMC12588454; doi:10.1371/journal.pntd.0013634)
Supplement: Attachment — Submitted filename: Reviewers Answers.pdf [file pntd.0013634.s003.pdf]

We would like to thank to all the reviewers for their comments and we did our best to make all the suggested changes possible and to reply to each comment individually. Replies to the general comments below.

To the general comments:
The author summary was added.
The authors' contribution section was added.
The trademark symbols were removed.
All the pictures (1, 2 and 3) were obtained by authors during the fieldwork.
The maps were created in ArcGis software with Natural Earth - All maps are public domain.
The role in the study design statement was added to the Financial disclosure statement - section "Funding" at the end of the document.
Conclusion section was added.

Reviewer 1

I suggest that the authors detail the methodology used for each stage of this study, as it can be reproduced by other researchers.
We did our best to rewrite the Methodology section so it is clearer and reproducible

The manuscript is original, it is well written and presents interesting data on the malacological surveillance of natural hosts of A. cantonensis and reinforces Achatina fulica as the intermediate host that most favors the maintenance of the parasite cycle in the environments studied. This has been demonstrated in several countries with a large dispersion of this snail, such as in Brazil. Some other references can be added. I suggest that the authors detail the methodology used for each stage of this study, as it can be reproduced by other researchers. In their results, they could include a comparison between the results of the LAMP and qPCR assays. Both were performed, if I understood correctly. Did any sample present different results between them? Please make this point clearer.
Thank you for the suggestions, we did our best to make both Methods and Results sections of LAMP and qPCR clearer and reproducible. Since LAMP was done on pools only and qPCR on individuals from positive LAMP pools, the suggested comparison is not possible, but we changed the text so it is obvious which assay resulted positive and negative.

In their results, they could include a comparison between the results of the LAMP and qPCR assays. Both were performed, if I understood correctly. Did any sample present different results between them? Please make this point clearer.
We changed the text accordingly.

Pg 1 line 16 - Lissachatina fulica – Lissachatina is a subgenus: Achatina (Lissachatina) fulica. There is not enough phylogenetic support to elevate it to a genus. Many authors have used it as a genus in recent years, which causes a lot of confusion. I suggest changing it throughout the text. The subgenus should be cited only at the beginning of the text. After that, use only "A. fulica"
Corrected in the text
Pg 1 Line 28 – abbreviate A. cantonensis after first mention in the text
Corrected
Pg 1 Line 45 – Please cite examples of freshwater vertebrates

Corrected

Pg 3 Line 61 – Please describe the LAMP assay to help the reader understand better. But only do so in the next topic. How was this pooled analysis performed? What steps were taken? And how much of each mollusk was analyzed?

In the topic "Sample collection (52-76)", I suggest separating the information that actually deals with the collection and, only then, talking about the procedures for parasitological analysis of each group of mollusks. I believe it will be clearer this way.

The next topic deals with molecular analyses. In it, the authors can talk about the LAMP method.

Thank you for the suggestions. We changed the text accordingly and did our best to make the section clearer.

Pg 3, Line 108 – I suggest starting with "The terrestrial snail A. fulica…".

Corrected

Pg 4, Lines 119-121: I suggest: Five DNA sequences of A. cantonensis recovered from A. fulica samples were obtained from two different localities (City Center - park and Nurul Qualbi gardens) ranging from 322 to 554 bp.

Corrected

Reviewer 2

Pls provide the primer sequences of ITS1 with thermal cycle of the ITS1 based PCR

Thank you for the comment, the ITS1 targeted PCR is not originally designed for the purpose of this manuscript, we used the primers and cycling conditions as in Izquierdo-Rodriguez et al., 2023 - as stated in the Methods section. We changed the text so it clearly states which primers were used.

Reviewer 3

- "Notably, the handling and consumption of snails sold at wet markets do not appear to increase the risk of eosinophilic meningitis in this region." May this be because the freshwater snails selled in the wet market do not come from this area? Consider adding some info regarding this point in the discussion. Maybe Sulcospira species from the market are not positive because they come from other areas luckily free of cantonensis. Just an idea, but stating that it do not contribure… well, in this case. Has Sulcospira being reported as IH in other regions? If not, then it might not be a good IH, independently of being selled in the wet market.

Thank you for the suggestion. We changed the text accordingly and explained better in the discussion.

L6: references mention only a few specific cases of the rat lungworm in wildlife. If not reporting all the species reported as accidental, I recomment to reduce the references to the two reviews (ref. 4 and 6). All animal groups mentioned in the rest of references appear in those two reference. In fact, reference 5 is included in the review of the 4…

References were deleted from the text.

L13: Please, put the scientific names of the rat species found in Sumatra in brackets "… also identified in rats (Rattus tiomanicus…. and Rattus…).

Corrected

L15: add here the common name of Lissacatina fulica and delete it from line 19.

We have changed the text so it is clear Line 15 is not referring only to A. fulica (therefore the common name has to remain in Line 19)

L16: Add the S of the plural "South Sumatra ProvinceS".
Corrected
L19: I would suggest unifying all the snail's species photographs in a single figure containing the three photos at once. This way it is easier to follow and have a general idea on the species involved in the study.
The images were merged into one figure.
L22: "the abundance of these gastropods in Aceh province is high". Please, add a reference for this affirmation.
The references were added.
L26: As mentioned above, unify all snail photos in a single figure.
Corrected

I am missing an important part here, the identification of the snails. Although I understand these species may not be difficult to identify, being this study focused on the snails, the correct identification of each genus mentioned is crucial and should be confirmed. Please, add the references used for the identification of the snails. If non used, consider performing some PCR of a few individuals per genus to be sure it is correct. Add a small section with all the methods.
Thank you for the suggestion. In our team, a malacologist determined A. fulica and Pomacea sp., common invasive species - which identification is trivial for an experienced malacologist. In the case of Sulcospira, we added a reference for morphological identification that was used. The reference deals with gastropods in Vietnam, however the studies from Sumatra are missing - and that is why we only determined the Sulcospira specimens only to a genus level.

L62: Why was LAMP used and then qPCR instead of performing just the qPCR? Explain.
We clarified in the text - Methods - LAMP section.
L69: Why was the additional procedure of the emerging of Angiostrongylus performed in these snails instead of analysing just the tissue snips as in Lissachatina? Are we able to compare results using different methods?
We did our best to clarify this in the text - methods as well as below (comment to the Line 143)
L75: Now the Sulcospira (also an aquatic snail) samples are analysed directly without doing the emergence of larvae step. Why is this? Why not performing the same analysis in all snails the same way? Results may differ because of the techniques. Please, clearly explain the reason to differ in the methodology per species.
We did our best to clarify this in the text - methods as well as below (comment to the Line 143)
The preferred method - the one used in Achatina and Sulcospira, could not be performed in Pomacea - see below. But since the aim is to detect DNA of the parasite, it is highly unlikely to miss the DNA if even a larval fragment is present (Sears et al. 2021)
L80: I assume you wanted to say "99ºC".

Corrected

L85: “with modification optimized for L3 larvae of A. cantonensis, when the pre-lyse phase was extended overnight”. I would rewrite it, as it seems not natural to me using that “when”. Maybe something like “extending the pre-lyse phase overnight as a modification for L3 larvae of A. cantonensis.

I would rewrite the whole Methods section; it is not clear if LAMP was performed in all samples or just in Lissachatina and Pomacea snails. Also, there is different methodology per genus without explanation.

We clarified in the text and changed the text accordingly.

L109: Please, give 95% Confidence Interval (exact method) together with the prevalence (add this in the section methods), and a bit on the descriptive analysis.

We changed the text accordingly.

L104: Please, delete the blank space between 6._7% (6.7%).

Corrected

L117: Fig 3 refers to a photograph of the Sulcospira snails. Do you mean the map of the Fig. 4? Please check. Also, important, ADD scale bar to maps.

The reference was corrected, and a scale bar was added to the map.

L119: I suggest writing “five DNA sequences varying from 322 to 554 bp from …”. In this section I miss a clear result on the LAMP and posterior confirmation of the qPCR. Only final positives are reported, but it would be interesting to know if all LAMP positives were confirmed with qPCR or if there were some false positive pools.

We changed the text accordingly.

L141: “Notably, both A. cantonensis and A. malaysiensis are zoonotic.”. Why this sentence here?

This sentence was removed from the main text

L143: Are we sure on the absence of A. cantonensis in this species as we performed a different methodology?

We applied a different processing method for *Pomacea* sp. compared to other snail species due to anatomical constraints. *Pomacea* snails possess extremely hard shells and a tight-closing operculum, making dissection difficult and potentially hazardous without heavy mechanical force. Crushing them in hard zip-lock bags partially filled with water allowed us to (1) break the shells effectively, (2) pool and process specimens efficiently, and (3) promote larval emergence and sedimentation, as previously described by Modrý et al. (2021). This method is highly sensitive - qPCR assays can detect as little as 1/100,000 of a larva (Sears et al. 2021) - and therefore, we consider it highly unlikely that *A. cantonensis* DNA would have been missed in positive individuals using this approach.

We have changed the text so it is clear why we chose this approach.

L160: I suggest adding these two references together with ref. 19: https://doi.org/10.1371/journal.pone.0094969 and https://doi.org/10.1016/j.onehlt.2023.100610.

The suggested references deal with intermediate hosts of the parasite while the sentence and our provided references are about paratenic hosts.
L166-168: Might this also be driven by rat distribution and densities?
This note was added to the main text
L173: Start the sentence with a “the”. The presence.
Corrected

Maybe a small sentence regarding why you do not differenciate between snail species in this study would be great. Consider adding something similar in the methods’ section.
Clarified in the text.
Ref. 29: Italics in scientific name.
Corrected

Figure 1-3: As mentioned above in the text, it would be clear to have the three snail photos in the same figure. Also, apart from the photo of L. fulica, the rest of the species are not clearly visible. Although these are good pictures of the snails in the environment and in the wet market, consider adding some photos with a clear view of the snail to see the morphology a bit more, especially when no info on the snails’ identification has been provided in the text.
The figures were merged into one. We provided a reference according to which Sulcospira was identified.
Figure 4: Add scale, at least in the zoomed map.
Scale bar was added to the map.
