# Supplementary material for: Hidden threats in urban environments: Angiostrongylus cantonensis in Banda Aceh’s cityscape
Source: PLoS Negl Trop Dis. 2025 Oct 30;19(10):e0013634. doi: 10.1371/journal.pntd.0013634 (PMC12588454; doi:10.1371/journal.pntd.0013634)
Supplement: Attachment — Submitted filename: PNTD-D-25-00434_R1 08_2025 08_2025 Rev.pdf [file pntd.0013634.s004.pdf]

PLOS Neglected Tropical Diseases

Hidden Threats in Urban Environments: Angiostrongylus cantonensis in Banda Aceh's Cityscape

--Manuscript Draft--

**Manuscript Number:**	PNTD-D-25-00434R1
**Full Title:**	Hidden Threats in Urban Environments: Angiostrongylus cantonensis in Banda Aceh's Cityscape
**Short Title:**	Hidden Threats in Urban Environments: Angiostrongylus cantonensis in Banda Aceh
**Article Type:**	Research Article
**Keywords:**	Angiostrongylus cantonensis, Rat Lungworm, Public Health, Snails, Food-borne Nematode
**Abstract:**	Angiostrongylus cantonensis is a mollusk-borne parasitic nematode originating in Southeast Asia. Commonly known as the rat lungworm, it uses rats as definitive hosts, though other mammals, including humans, can be infected and typically suffer from neurological disorders. This study focuses on the parasite's presence in its gastropod intermediate hosts in several urban and rural areas in Aceh province, Sumatra, Indonesia. Samples of Achatina (Lissachatina) fulica (161) and Pomacea sp. (90) were collected in eight localities in Banda Aceh. Additionally, 531 edible freshwater snails belonging to the genus Sulcospira sp. from three different localities in Aceh province were obtained in wet markets. All samples were examined by LAMP and qPCR for the A. cantonensis DNA. No samples of Sulcospira sp. and Pomacea sp. tested positive. 13.4% of L. fulica tested positive, with the highest prevalence in urban areas of Banda Aceh. The ITS1 sequences obtained from positive samples using conventional PCR<br>confirmed 100% identity with A. cantonensis. The present study confirms, for the first time, the presence of the zoonotic parasite A. cantonensis in Banda Aceh, Sumatra, Indonesia. Notably, the handling and consumption of snails sold at wet markets do not appear to increase the risk of eosinophilic meningitis in this region. However, the relatively high prevalence of A. cantonensis in urban land snails underscores the need for continued vigilance and public health awareness.
**Additional Information:**	
**Question**	**Response**
**Government Employee**<br><br>Are you or any of the contributing authors an employee of the United States government?<br><br>Manuscripts authored by one or more US Government employees are not copyrighted, but are licensed under a CC0 Public Domain Dedication, which allows unlimited distribution and reuse of the article for any lawful purpose. This is a legal requirement for US Government employees.<br><br>This will be typeset if the manuscript is accepted for publication.	No - No authors are employees of the U.S. government.

Powered by Editorial Manager® and ProduXion Manager® from Aries Systems Corporation

**Financial Disclosure**<br><br>Enter a financial disclosure statement that describes the sources of funding for the work included in this submission. Review the submission guidelines for detailed requirements. View published research articles from *PLOS Neglected Tropical Diseases* for specific examples.<br><br>This statement is required for submission and **will appear in the published article** if the submission is accepted. Please make sure it is accurate.<br><br>**Funded studies**<br>Enter a statement with the following details:<br>• Initials of the authors who received each award<br>• Grant numbers awarded to each author<br>• The full name of each funder<br>• URL of each funder website<br>• Did the sponsors or funders play any role in the study design, data collection and analysis, decision to publish, or preparation of the manuscript?<br><br>Did you receive funding for this work?	Yes
Please add funding details.<br>as follow-up to "**Financial Disclosure**<br><br>Enter a financial disclosure statement that describes the sources of funding for the work included in this submission. Review the submission guidelines for detailed requirements. View published research articles from *PLOS Neglected Tropical Diseases* for specific examples.<br><br>This statement is required for submission and **will appear in the published article** if the submission is accepted. Please make sure it is accurate.	This work was supported by the SEAEUROPEJFS19IN-053 project and the Czech Science Foundation grant no. 22-26136S (both to DM). The work of Lucia Anettová (LA) and Anna Šipková (AŠ) was also supported by Specific Research – support of student projects, no. MUNI/A/1602/2023. The funders had no role in study design, data collection and analysis, decision to publish, or preparation of the manuscript.

**Funded studies**<br>Enter a statement with the following details:<br>• Initials of the authors who received each award<br>• Grant numbers awarded to each author<br>• The full name of each funder<br>• URL of each funder website<br>• Did the sponsors or funders play any role in the study design, data collection and analysis, decision to publish, or preparation of the manuscript?<br><br>Did you receive funding for this work?"	
Please select the country of your main research funder (please select carefully as in some cases this is used in fee calculation).<br>as follow-up to "**Financial Disclosure**<br><br>Enter a financial disclosure statement that describes the sources of funding for the work included in this submission. Review the submission guidelines for detailed requirements. View published research articles from *PLOS Neglected Tropical Diseases* for specific examples.<br><br>This statement is required for submission and **will appear in the published article** if the submission is accepted. Please make sure it is accurate.<br><br>**Funded studies**<br>Enter a statement with the following details:<br>• Initials of the authors who received each award<br>• Grant numbers awarded to each author<br>• The full name of each funder<br>• URL of each funder website<br>• Did the sponsors or funders play any role in the study design, data collection and analysis, decision to publish, or preparation of the manuscript?<br><br>Did you receive funding for this work?"	CZECH REPUBLIC - CZ
**Competing Interests**	The authors have declared that no competing interests exist

On behalf of all authors, disclose any competing interests that could be perceived to bias this work.<br><br>This statement will be typeset if the manuscript is accepted for publication.<br><br>Review the instructions link below and PLOS NTDs' competing interests policy to determine what information must be disclosed at submission.	
**Data Availability**<br><br>Provide a **Data Availability Statement** in the box below. This statement should detail where the data used in this submission can be accessed. This statement will be typeset if the manuscript is accepted for publication.<br><br>Before publication, authors are required to make all data underlying their findings fully available, without restriction. Review our PLOS Data Policy page for detailed information on this policy. Instructions for writing your Data Availability statement can be accessed via the Instructions link below.	All data generated or analyzed during this study are included in this published article.

Manuscript

Click here to access/download;Manuscript;Aceh_MS_PLoS 22.7.2025_MAIN_TEXT.pdf

Hidden Threats in Urban Environments: *Angiostrongylus cantonensis* in Banda Aceh's Cityscape

Lucia Anettová[1,2◕]*, Anna Šipková[2◕], Vojtech Baláž[3], Muhammad Hambal[4], Radovan Coufal[2], Jana Kačmaříková[5], Henni Vanda[4], Wahyu Eka Sari[4], David Modrý[1,2,6]

1 Department of Veterinary Sciences/Faculty of Agrobiology, Food and Natural Resources, Czech University of Life Sciences, Prague, Czech Republic
2 Department of Botany and Zoology/Faculty of Science, Masaryk University, Brno, Czech Republic
3 Department of Ecology and Diseases of Zoo Animals, Game, Fish and Bees, Faculty of Veterinary Hygiene and Ecology, University of Veterinary Sciences Brno, Czech Republic
4 Faculty of Veterinary Medicine/University Syiah Kuala, Banda Aceh, Indonesia
5 Department of Pathology and Parasitology/Faculty of Veterinary Medicine, University of Veterinary Sciences Brno, Czech Republic
6 Institute of Parasitology/Biology Center/Czech Academy of Sciences, České Budějovice, Czech Republic

◕These authors contributed equally to this work.
* lucia.anettova@gmail.com

Abstract

Angiostrongylus cantonensis is a mollusk-borne parasitic nematode originating in Southeast Asia. Commonly known as the rat lungworm, it uses rats as definitive hosts, though other mammals, including humans, can be infected and typically suffer from neurological disorders. This study focuses on the parasite's presence in its gastropod intermediate hosts in several urban and rural areas in Aceh province, Sumatra, Indonesia. Samples of *Achatina* (*Lissachatina*) *fulica* (161) and *Pomacea* sp. (90) were collected in eight localities in Banda Aceh. Additionally, 531 edible freshwater snails belonging to the genus *Sulcospira* sp. from three different localities in Aceh province were obtained in wet markets. All samples were examined by LAMP and qPCR for the *A. cantonensis* DNA. No samples of *Sulcospira* sp. and *Pomacea* sp. tested positive. 13.4% of *L. fulica* tested positive, with the highest prevalence in urban areas of Banda Aceh. The ITS1 sequences obtained from positive samples using conventional PCR confirmed 100% identity with *A. cantonensis*. The present study confirms, for the first time, the presence of the zoonotic parasite *A. cantonensis* in Banda Aceh, Sumatra, Indonesia. Notably, the handling and consumption of snails sold at wet markets do not appear to increase the risk of eosinophilic meningitis in this region. However, the relatively high prevalence of *A. cantonensis* in urban land snails underscores the need for continued vigilance and public health awareness.

Author Summary

Angiostrongylus cantonensis, commonly known as rat lungworm, is a parasitic nematode that can cause serious neurological disease in humans and other accidental hosts. It is transmitted via snail or slug intermediate hosts, typically through accidental ingestion

of infected snails or contaminated produce. In this study, we investigated the presence of *A. cantonensis* in land and freshwater snails from urban and rural areas of Aceh province, Sumatra, Indonesia — region where its occurrence had not previously been documented. We tested over 780 snails using molecular diagnostic techniques and found that *Achatina* (*Lissachatina*) *fulica*, an invasive land snail commonly found in urban environments, had a relatively high infection rate. In contrast, none of the freshwater snails sold for consumption at local wet markets tested positive. Our findings represent the first molecular confirmation of *A. cantonensis* in Banda Aceh. While current practices around the handling and consumption of freshwater snails may pose little risk in this area, the presence of the parasite in urban land snails highlights the importance of public health awareness and monitoring, especially in densely populated settings where contact with infected snails is more likely.

Introduction

The rat lungworm *Angiostrongylus cantonensis* is a food-borne zoonotic metastrongyloid nematode first described in Canton, China (1). However, it has expanded throughout the world and is considered an emerging pathogen (2), causing eosinophilic meningitis in humans and similar neurological disorders in numerous mammalian and avian species considered aberrant hosts (3; 4).

Cases of human eosinophilic meningitis likely caused by *A. cantonensis* have been reported from Southeast Asia since the 1960s (5; 6). The parasite's presence in both, definitive and intermediate hosts or in humans (as its accidental hosts) have been confirmed on most of the main islands of Indonesia (6; 7; 8; 9). In Sumatra, eight human cases of eosinophilic meningitis likely caused by *A. cantonensis*, were first reported in Kisaran, North Sumatra Province (10). The parasite was also identified in rats (*Rattus tiomanicus jalorensis* and *Rattus rattus diardii*), originating from the surroundings of Medan, North Sumatra Province (11). Although no further human infections have been documented, the parasite has been detected in rats and snails (including *A. fulica*) across the island, particularly in South Sumatra Provinces (Lubuk Linggau, Baturaja, and Martapura) and Lampung Province (7; 12; 13). However, the northwestern part of Sumatra remains largely unstudied.

The giant terrestrial African snail (*A. fulica*, Fig 1A) and the freshwater apple snail (*Pomacea* spp., Fig 1B) are among notable invasive mollusks associated with the zoonotic transmission of *A. cantonensis* to humans (2; 14; 15). Resulting from a successful invasion, the abundance of these gastropods in Aceh province is high (16; 17) and these species could serve as suitable terrestrial and aquatic intermediate hosts for *A. cantonensis* in the area. Although widely distributed in Aceh province, these mollusks are generally not used for human consumption, in contrast to freshwater *Sulcospira* spp. (Fig 1C) commonly consumed by the local population. However, the potential role of this species as an intermediate host for *A. cantonensis* remains undetermined.

Rats of the genus *Rattus* are definitive hosts for *A. cantonensis*, with gastropods serving as intermediate hosts. The parasite's life cycle involves first-stage larvae (L1) excreted in rat feces, developing into infective third-stage larvae (L3) within gastropods (18). Poikilothermic animals (including reptiles and amphibians) can act as paratenic hosts (19; 20; 21; 22), transmitting infection to humans and other mammals. Due to the invasion and spread of the parasite, facilitated by rats and gastropods, resulting disease is becoming increasingly common in humans and is considered one of the emerging infectious diseases (2).

Apart from direct ingestion of intermediate or paratenic hosts, food, water or hands contaminated with free L3 released from gastropods represent another route of transmission to humans (18; 23). In the human body, the parasites reach the brain in

the same way as in rats and develop to the subadult stage. The immune reaction and the presence of larvae in the brain result in severe neurological symptoms, occasionally with fatal consequences (24).

Aceh Province is the northernmost part of Sumatra, characterized by a warm and humid climate that is conducive to the life cycle of *A. cantonensis*. Culinary habits in Aceh include the consumption of *Sulcospira* snails as well as other freshwater invertebrates (such as crustaceans) and vertebrates (e.g., fish), which are potential paratenic hosts for *A. cantonensis* (22), underscoring the need for ongoing monitoring and public health vigilance. Our study investigates the presence of *A. cantonensis* in intermediate hosts in urban, suburban and rural areas of Banda Aceh and potential risk for zoonotic infections. To the authors´ knowledge, this is the first study to investigate the presence of *A. cantonensis* in Aceh province.

Materials and methods

Sample collection

Landsnails *A. fulica* were collected in five localities, two urban: city center (1) and campus of the University Syiah Kuala (2); one suburban: Lambirah (3); and two rural: pastures near Kuede (4) and gardens by Nurul Qualbi (5). The urban locality in the city center was further divided into two sublocalities: park (1a) and riverside (1b) (Table 1, Fig 2). The localities were characterized as urban, suburban or rural based on land use and infrastructure in the particular areas, taking into account population density in the areas. In each locality/sublocality, at least 30 snails were collected, except for the locality 4, where we were able to find only 11 individuals. All collected *A. fulica* were euthanized by freezing.

Pomacea sp. were collected in three localities: two suburban (Lambirah and along Jl. Tgk. Bakurma) and one urban (University Syiah Kuala campus). Thirty snails were collected from each locality. A different methodology was applied for *Pomacea* sp. due to their extremely hard shells and tight-closing operculum, which make standard dissection impractical. Snails were crushed in sealed plastic bags filled with water (approx. 50 ml), allowing larvae to emerge and sediment for downstream molecular analysis, following the approach of Modrý et al. (25). After one hour, 0.5 ml of sediment was collected from each bag for DNA extraction using PrepMan, to be used in the LAMP assay.

In wet markets, edible freshwater snails *Sulcospira* sp. from three different localities in Aceh province were obtained. 150 snails were obtained from Lhoong, Aceh Besar District, 91 from Leupung Subdistrict, Aceh Besar District and 290 from Lamno, Aceh Jaya District. The snails from wet markets were treated the same way as *A. fulica*, examined in pools of 10 individuals. The snails were identified to the genus level based on Köhler et al. (26).

Both aquatic snails were identified morphologically to the genus level, due to the limited availability of studies on aquatic gastropods in Indonesia. The taxonomy of the genus *Sulcospira* in Sumatra remains unresolved, and the exact species present in the region are currently unknown. Specimens of *Pomacea* likely belong to either *P. canaliculata* or *P. maculata*, two morphologically similar species that can be reliably distinguished only through genetic analysis. In contrast, *A. fulica* is morphologically distinct and easily identifiable to the species level. All the gastropod species collected are shown in Fig 1.

Prevalence was calculated as the proportion of positive samples among the total number examined, and 95% confidence intervals were computed using the Clopper–Pearson exact method. The analysis was descriptive and focused on

summarizing prevalence across species and localities.

Molecular analysis and sequence data

LAMP

All samples were initially screened using the LAMP assay due to its lower cost and simpler workflow compared to qPCR, allowing for rapid elimination of negative pools and more efficient processing. In case of *A. fulica*, pools of three individuals were used, using approximately 10 µg of a foot tissue from each snail. Tissue samples from each individual were also stored for further qPCR analysis in case the pooled LAMP analysis was positive. First, DNA was extracted from snail tissues using PrepMan Ultra Sample Preparation Reagent (Thermo Fisher Scientific, Waltham, MA, USA), according to the manufacturer's instructions. Briefly, PrepMan and tissue were used in a 1:1 ratio in 2mL vials, i.e., 50–150 µL of PrepMan was added depending on the tissue amount. The mixture was incubated at 99 °C in a water bath for 10 minutes and used immediately. The LAMP assays were performed using the Genie II platform (OptiGene, UK) in 20 µL reactions, as described by Baláž et al. (27). In case of *Pomacea* sp. and *Sulcospira* sp., pools of three and 10 individuals respectively were processed by the LAMP in the same way as the samples of *A. fulica*.

qPCR

Pooled samples of *A. fulica* which tested positive with LAMP subsequently underwent the species specific qPCR. DNA from all the individuals in pools that tested positive by LAMP was subsequently extracted using DNEasy Blood&Tissue (Qiagen, Germany), extending the pre-lyse phase overnight as a modification for L3 of *A. cantonensis*. Approximately 25 µg of foot tissue was used. The qPCR assay was performed on BIO-RAD CFX96 Real Time system in a 20 µL reaction, according to Sears et al. (28) and modified as in Anettová et al. (29). As positive controls, DNA from a single L3 of *A. cantonensis* extracted by the same method as samples and diluted 100x was used.

Additionally, SYBR Green-based quantitative real-time PCR as in Jakkul et al. (30) was performed on the DNA samples tested positive previously on qPCR to investigate possible co-occurence of *A. malaysiensis*. The assay was run in duplicates and two sets of primers for the two nematodes were used. In case of confirmation of *A. cantonensis* the primers were used as originally published. In case of *A. malaysiensis*, we have adjusted the length of primers, to cover more variable positions and increase annealing temperature. The primers used in SYBR Green qPCR can be found in Table 2.

Conventional PCR

To confirm the identity of larvae as *A. cantonensis* in *A. fulica*, nested PCR was performed to obtain ITS1 sequences from the snail individuals that tested positive on qPCR. This involved using the ITS1-Canto-F3 and ITS1-Canto-R1 primers targeting the ITS-1 region of *A. cantonensis*, following the PCR cycling conditions described by Izquierdo-Rodriguez et al. (31), from which these primers were originally adopted. PCR products were purified with ExoSAP-IT (Affymetrix, ThermoFisher Scientific, Czech Republic) and sequenced commercially at Macrogen Europe (Amsterdam, Netherlands) using the amplification primers. Sequences were assessed and trimmed manually in Geneious Prime 2024.0.5 (http://www.geneious.com) and checked against BLAST (32).

1 Results

Of the 55 pools of *A. fulica*, 12 tested positive. All pools that were positive by LAMP (each consisting of three *A. fulica* individuals) were confirmed by qPCR, with at least one individual in each pool testing positive. The terrestrial snail *A. fulica* was the only species that tested positive for *A. cantonensis* DNA on both, LAMP and qPCR. Of the 164 specimens collected in Banda Aceh municipality and its surroundings, 22 (13.4%, 95% CI: 8.6–19.4%) tested positive. The highest prevalence was observed in the city center sublocalities: 6 of 32 samples (18.8%; sublocality 1a) and 11 of 31 samples (35.5%; sublocality 1b). At campus of University Syiah Kuala, 3 of 30 samples (10%) were positive, with one additional sample yielding dubious result. Among rural localities, only Nurul Qualbi (Loc 5) showed positive results, with 2 of 30 samples (6.7%) positive. No positive samples were found in Lambirah and Kuede. Aquatic snail *Sulcospira* sp. tested negative for *A. cantonensis* DNA in all examined localities by both assays, LAMP and qPCR. The results of *A. fulica* examination are summarized in Table 1; examples of positive and negative localities are shown in Fig 2. Since none of the aquatic snail pools tested positive by LAMP, no further analysis by qPCR was performed.

Five DNA sequences of *A. cantonensis* (BLAST analysis showed 100% identity with GenBank sequences of *A. cantonensis*) recovered from *A. fulica* samples were obtained from two different localities (City Center - park and Nurul Qualbi gardens) varying from 322 to 554 bp. The sequences obtained were identical, and one representative sequence from each locality has been submitted to GenBank with accession numbers PV133028 and PV133029. All DNA samples analyzed using SYBR Green-based real-time PCR tested positive with primers specific for *A. cantonensis*, while none tested positive for *A. malaysiensis.*

Discussion

The study provides further data on distribution of *A. cantonensis* in Sumatra (10; 11), with first sequence data from this region. Our findings confirm the presence of *A. cantonensis* in the municipality of Banda Aceh, with DNA detected only in the terrestrial African giant snail *A. fulica* with a remarkable prevalence, especially in the city center. Conversely, all examined samples from aquatic gastropods tested negative, despite a relatively large sample size of edible aquatic snails, including *Sulcospira* sp. and *Pomacea* sp. These results suggest absence or a very low presence of *A. cantonensis* in the aquatic environment in tested localities of the Aceh province.

To confirm the taxonomic identity of the nematode and to exclude possible co-occurence with *A. malaysiensis* as *A. cantonensis*, ITS1 sequence were analyzed and the SYBR-Green based real-time PCR was conducted, providing an ultimate taxonomic identification. While the qPCR assay used is highly sensitive and specific (28), it is not suitable for distinguishing among the three sibling species: *A. cantonensis*, *A. malaysiensis* (which likely overlaps with *A. cantonensis* in Southeast Asia), and *A. mackerrasae* (found in Australia).

The absence of *A. cantonensis* in *Pomacea* sp. in areas where *L. fulica* tested positive was unexpected. This disparity may indicate low exposure of aquatic mollusks in tested localities to *A. cantonensis* larvae or that *Pomacea* sp. are less suitable intermediate hosts compared to *A. fulica.* Previous studies support the latter hypothesis, showing lower prevalence in *Pomacea* spp. compared to terrestrial gastropods such as *A. fulica* (33). However, no experimental studies have directly compared the suitability of these two intermediate hosts for supporting *A. cantonensis* larvae, highlighting a potential area for future research. Alternatively, this absence in aquatic hosts could also

Table 1. Sampling localities of *Achatina* (*Lissachatina*) *fulica* in Banda Aceh and its surroundings, with the categorization and numbers of samples tested positive by LAMP and qPCR.

Locality	Type	No. of Samples	No. Positive
(1a) City center (park)	urban	32	6
(1b) City center (riverside)	urban	31	11
(2) University Syiah Kuala	urban	30	3
(3) Lambirah	suburban	30	0
(4) Kuede	rural	11	0
(5) Nurul Qualbi gardens	rural	30	2

indicate an early stage of introduction in Aceh or environmental factors that limit the parasite's spread and development in these hosts and habitats. Further studies are needed to explore these factors and their influence on host-parasite dynamics.

Sulcospira sp., an edible aquatic snail frequently consumed as a traditional protein source in Aceh, tested negative for *A. cantonensis* DNA. To the authors' knowledge, this species has not previously been confirmed as an intermediate host. Several factors may explain the absence of *A. cantonensis* in the examined samples. First, all edible aquatic snails analyzed in this study originated from areas outside the Banda Aceh municipality, in contrast to the urban land snails (*A. fulica*) that tested positive. Second, although *A. cantonensis* is known for its low host specificity and ability to infect a wide range of gastropod species (18), it remains possible that *Sulcospira* sp. has a lower susceptibility or is less suitable as an intermediate host. Third, the aquatic snails were sourced exclusively from three extensive snail farms, which may represent simplified ecological environments with limited exposure to the full parasite life cycle — particularly if rat populations, essential for maintaining transmission, are sparse or absent.

Despite the negative results, the potential role of edible aquatic snails in transmission should not be underestimated. Handling and processing practices may still pose a risk of infection, especially if snails are undercooked or contaminated during transport or preparation. These findings underscore the importance of further investigations into aquatic hosts of *A. cantonensis* in the region, particularly under natural or semi-natural conditions, to better understand the transmission dynamics and potential risks to public health.

Our results emphasize predominant association of *A. cantonensis* with urban areas and its much lower prevalence in suburban and rural regions. This pattern may be driven by higher population densities of *A. fulica* in urban environments (34; 35), as this highly invasive species has a lower chance to be introduced directly into the natural or rural environment. However, as the snails and the parasite continue to spread, they are likely to eventually colonize these areas and find the environment suitable. Demonstrated presence in urban environment underscores risk of possible transmission via contaminated food or water.

The presence of *A. cantonensis* in this newly recognized area highlights the need for public health interventions, such as minimizing human contact with terrestrial snails, particularly in urban settings. Given the frequent consumption of freshwater gastropods and other aquatic hosts in Aceh, the potential for infection via aquatic pathways cannot be ignored. Further research should explore the prevalence of *A. cantonensis* in these hosts to assess the risk posed by local dietary practice (36; 37). Neuroangiostrongyliasis remains a significant public health concern and increased awareness is essential to mitigate its impact. Finally, the existence of underdiagnosed cases of eosinophilic meningitis in the region, particularly in areas with limited healthcare resources, should be considered (2; 38).

Table 2. Primers used in SYBR Green qPCR according to Jakkul et al. (30), with primers for *A. malaysiensis* modified

	A. cantonensis	*A. malaysiensis*
Forward	AC4_cytb_F (5′ AAT GTT TGT TGA GGC AGA TC 3′)	AM_cytb_Fnew (5′ CGA GAT ATT TAT TGA GGC TGA TCC TTT GAC 3′)
Reverse	AC5_cytb_R (5′ GCT ACA ACA CCC ATA ACC T 3′)	AM_cytb_Rnew (5′ GAC AAA ACC CTC ATC AAT AAA GCC A 3′)

2 Conclusion

This study provides the first molecular evidence of *A. cantonensis* in Banda Aceh, Sumatra, with high prevalence detected in *A. fulica*, particularly in urban areas. The absence of the parasite in examined aquatic gastropods, including *Pomacea* sp. and *Sulcospira* sp., suggests either low environmental exposure or limited suitability of these species as intermediate hosts. The findings underscore the potential public health risk in urban settings and highlight the need for increased surveillance, public education, and further research on alternative transmission pathways and host-parasite dynamics in both terrestrial and aquatic environments.

Authors' Contribution

Conceptualization: DM and MH. Data Curation: LA, AŠ and VB. Formal Analysis: VB, LA, RC, Funding Acquisition: DM and MH. Investigation: LA, AŠ, RC and VB. Methodology: LA, DM, AŠ, VB. Project Administration: DM, MH, WEH and HV. Resources: MH, WEH, HV. Suprevision: DM. Validation: DM, LA, VB. Visualization: AŠ, RC, DM. Writing – Original Draft Preparation: LA, AŠ. Writing – Review & Editing: DM, MH, RC, VB, LA, AŠ. All authors contributed critically to the drafts and gave final approval for publication.

Acknowledgments

The authors thank Petr Janoš for creating the map of localities studied for the presence of *Angiostrongylus cantonensis* in *Achatina* (*Lissachatina*) *fulica* (Fig 2).

Financial Disclosure

This work was supported by the SEAEUROPEJFS19IN-053 project and the Czech Science Foundation grant no. 22-26136S (both to DM). The work of Lucia Anettová (LA) and Anna Šipková (AŠ) was also supported by Specific Research – support of student projects, no. MUNI/A/1602/2023. The funders had no role in study design, data collection and analysis, decision to publish, or preparation of the manuscript.

Conflicts of Interest

The authors declare there are no conflicts of interest.

Figure Legend

Fig 1 Species of gastropods examined for the presence of *Angiostrongylus cantonensis* larvae in Aceh province, Indonesia (A) Invasive landsnail *Achatina* (*Lissachatina*) *fulica* found in the campus of the University Syiah Kuala, Banda Aceh; (B) Invasive aquatic snail *Pomacea* sp. found in a pond of the rural area near Banda Aceh; (C) Edible aquatic snail *Sulcospira* sp. from wet markets, originally from extensive snail farms in Aceh province. Photographs taken by RC and DM.

Fig 2 Sampling localities of *Achatina* (*Lissachatina*) *fulica* for the detection of *Angiostrongylus cantonensis* DNA in Banda Aceh, Indonesia. The map was created by the authors using ArcGIS and base layers from Natural Earth (http://www.naturalearthdata.com). All base layers are in the public domain and freely available under the terms of use specified at: http://www.naturalearthdata.com/about/terms-of-use/.

References

1. Chen HT. Un nouveau nématode pulmonaire, *Pulmonema cantonensis*, ng, n. sp. Annales de Parasitologie humaine et comparée. 1935;13(4):312–317.
2. Cowie RH, Ansdell V, Dunavan CP, Rollins RL. Neuroangiostrongyliasis: global spread of an emerging tropical disease. The American Journal of Tropical Medicine and Hygiene. 2022;107(6):1166.
3. Kim D, Stewart T, Bauer R, Mitchell M. *Parastrongylus* (= *Angiostrongylus*) *cantonensis* now endemic in Louisiana wildlife. Journal of Parasitology. 2002;88(5):1024–1026.
4. Spratt DM. Species of *Angiostrongylus* (Nematoda: Metastrongyloidea) in wildlife: A review. International Journal for Parasitology: Parasites and Wildlife. 2015;4(2):178–189.
5. Jindrák K, Alicata JE. A case of parasitic eosinophilic meningo-encephalitis in Vietnam probably caused by *Angiostrongylus cantonensis*. Annals of Tropical Medicine and Parasitology. 1965;59:294–300.
6. Scrimgeour EM. Distribution of *Angiostrongylus cantonensis* in Papua New Guinea. Transactions of the Royal Society of Tropical Medicine and Hygiene. 1984;78(6):776–779. doi:10.1016/0035-9203(84)90017-8.
7. Carney WP, Stafford EE, Purnomo, Tanudjaja S. Angiostrongyliasis in Indonesia: additional geographic and host occurrence records. Southeast Asian Journal of Tropical Medicine and Public Health. 1978;9(4):516–519.
8. Uga S, Ono K, Kataoka N, Hasan H. Seroepidemiology of five major zoonotic parasite infections in inhabitants of Sidoarjo, East Java, Indonesia. Southeast Asian Journal of Tropical Medicine and Public Health. 1996;27(3):556–561.
9. Lim BL. Occurrence of *Angiostrongylus cantonensis* in rats around Kuching, Sarawak. Annals of Tropical Medicine & Parasitology. 1967;61(4):429–431. doi:10.1080/00034983.1967.11686510.
10. Smit AM. Eosinophilic meningitis at Kisaran (Indonesia) and problem of its aetiology. Bulletin de la Société de Pathologie Exotique. 1962;55:720–722.

11. Kwo E, Kwo I. Occurrence of *Angiostrongylus cantonensis* in Rats in North Sumatra, Indonesia. The Journal of Parasitology. 1968;54(3):537–537. doi:10.2307/3277078.

12. Liat BL, et al. Studies of *Angiostrongylus cantonensis* and a *Malaysiensis* of Rodents Hosts in Two Transmigration Schemes in South Sumatera. Indonesian Bulletin of Health Research. 1978;6(1). doi:10.22435/bpk.v6i1Mar.342.

13. Stafford EE, Sukeri S, Sutanti T. The bandicoot rat, a new host record for *Angiostrongylus cantonensis* in Indonesia. Southeast Asian Journal of Tropical Medicine and Public Health. 1976;1:41–44.

14. de Brito FC, Joshi RC. The Golden Apple Snail *Pomacea canaliculata*: A review on invasion, dispersion and control. Outlooks on Pest Management. 2016;27(4):157–163.

15. Silva GM, Thiengo SC, Sierpe Jeraldo VL, Rego MIF, Silva ABP, Rodrigues PS, et al. The invasive giant African land snail, *Achatina fulica* (Gastropoda: Pulmonata): global geographical distribution of this species as host of nematodes of medical and veterinary importance. Journal of Helminthology. 2022;96:e86. doi:10.1017/S0022149X22000761.

16. Hendarsih-Suharto, Marwoto RM, Heryanto, Mulyadi, Siwi SS. The golden apple snail, Pomacea spp., in Indonesia. In: Joshi RC, Sebastian LS, editors. Global Advances in Ecology and Management of Golden Apple Snails. Los Baños, Philippines: Philippine Rice Research Institute (PhilRice); 2006. p. 231–242.

17. Nurinsiyah AS, Hausdorf B. Listing, impact assessment and prioritization of introduced land snail and slug species in Indonesia. Journal of Molluscan Studies. 2018;85(1):92–102. doi:10.1093/mollus/eyy062.

18. Cowie RH. Pathways for transmission of angiostrongyliasis and the risk of disease associated with them. Hawaii Journal of Medicine & Public Health. 2013;72(6 Suppl 2):70–74.

19. Anettová L, Izquierdo-Rodriguez E, Foronda P, Baláž V, Novotný L, Modrý D. Endemic lizard *Gallotia galloti* is a paratenic host of invasive *Angiostrongylus cantonensis* in Tenerife, Spain. Parasitology. 2022;149(7):934–939.

20. Ash LR. The occurrence of *Angiostrongylus cantonensis* in frogs of New Caledonia with observations on paratenic hosts of metastrongyles. The Journal of Parasitology. 1968; p. 432–436.

21. Rosen L, Chappell R, Laqueur GL, Wallace GD, Weinstein PP. Eosinophilic meningoencephalitis caused by a metastrongylid lung-worm of rats. Jama. 1962;179(8):620–624.

22. Turck HC, Fox MT, Cowie RH. Paratenic hosts of *Angiostrongylus cantonensis* and their relation to human neuroangiostrongyliasis globally. One Health. 2022; p. 100426.

23. Šipková A, Anettová L, Izquierdo-Rodriguez E, Velič V, Modrý D. Release of *Angiostrongylus cantonensis* larvae from live intermediate hosts under stress. Parasitology Research. 2024;123(5):1–7.

24. Kliks MM, Palumbo NE. Eosinophilic meningitis beyond the Pacific Basin: the global dispersal of a peridomestic zoonosis caused by *Angiostrongylus cantonensis*, the nematode lungworm of rats. Social Science & Medicine. 1992;34(2):199–212. doi:10.1016/0277-9536(92)90097-a.

25. Modrý D, Fecková B, Putnová B, Manalo SM, Otranto D. Alternative pathways in *Angiostrongylus cantonensis* (Metastrongyloidea: Angiostrongylidae) transmission. Parasitology. 2021;148(2):167–173.

26. Kohler F, Holford M, Tu DV, Hai HT. Exploring a largely unknown fauna: On the diversity of pachychilid freshwater gastropods in Vietnam (Caenogastropoda: Cerithioidea). Molluscan Research. 2009;29(3):121–146. doi:10.11646/mr.29.3.1.

27. Baláž V, Rivory P, Hayward D, Jaensch S, Malik R, Lee R, et al. Angie-LAMP for diagnosis of human eosinophilic meningitis using dog as proxy: A LAMP assay for *Angiostrongylus cantonensis* DNA in cerebrospinal fluid. PLoS Neglected Tropical Diseases. 2023;17(5):e0011038.

28. Sears WJ, Qvarnstrom Y, Dahlstrom E, Snook K, Kaluna L, Baláž V, et al. AcanR3990 qPCR: a novel, highly sensitive, bioinformatically-informed assay to detect *Angiostrongylus cantonensis* infections. Clinical Infectious Diseases. 2021;73(7):e1594–e1600.

29. Anettová L, Kačmaříková J, Šipková A, Velič V, Cavallo L, Dyková I, et al. Low potential of fish as a source of infection with *Angiostrongylus cantonensis*. Food Quality and Safety. 2024; p. fyae042.

30. Jakkul W, Chaisiri K, Saralamba N, Limpanont Y, Dusitsittipon S, Charoennitiwat V, et al. Newly developed SYBR green-based quantitative real-time PCRs revealed coinfection evidence of *Angiostrongylus cantonensis* and *A. malaysiensis* in *Achatina fulica* existing in Bangkok metropolitan, Thailand. Food and waterborne parasitology. 2021;23:e00119.

31. Izquierdo-Rodriguez E, Anettová L, Hrazdilová K, Foronda P, Modrý D. Range of metastrongylids (superfamily Metastrongyloidea) of public health and veterinary concern present in livers of the endemic lizard Gallotia galloti of Tenerife, Canary Islands, Spain. Parasites & Vectors. 2023;16(1):81.

32. Altschul SF, Gish W, Miller W, Myers EW, Lipman DJ. Basic local alignment search tool. Journal of molecular biology. 1990;215(3):403–410.

33. Song L, Wang X, Yang Z, Lv Z, Wu Z. *Angiostrongylus cantonensis* in the vector snails *Pomacea canaliculata* and *Achatina fulica* in China: a meta-analysis. Parasitology Research. 2016;115:913–923.

34. Thiengo SC, Faraco FA, Salgado NC, Cowie RH, Fernandez MA. Rapid spread of an invasive snail in South America: the giant African snail, *Achatina fulica*, in Brasil. Biological Invasions. 2007;9:693–702.

35. Gamarra-Rueda R, García R, Restrepo-Rodas DC, Pérez-García J. First identification of *Angiostrongylus* spp. in *Lissachatina fulica* and *Cornu aspersum* in Antioquia, Colombia. Biomédica. 2024;44(3):416–424.

36. Ervilita R, Mardhiah A. Local Wisdom of the Aceh Jaya Society in terms of Indigenous Cuisine that can be learned in Chemistry Learning in Schools. In: Proceedings of International Conference on Multidiciplinary Research. vol. 5; 2022. p. 134–146.

37. Muchlisin ZA, Rinaldi F, Fadli N, Adlim M, Siti-Azizah MN. Food preference and diet overlap of two endemic and threatened freshwater fishes, depik (*Rasbora tawarensis*) and kawan (*Poropuntius tawarensis*) in Lake Laut Tawar, Indonesia. Aquaculture, Aquarium, Conservation & Legislation. 2015;8(1):40–49.

38. Pandian D, Najer T, Modrý D. An overview of *Angiostrongylus cantonensis* (Nematoda: Angiostrongylidae), an emerging cause of human angiostrongylosis on the Indian subcontinent. Pathogens. 2023;12(6):851.

Fig 1

Click here to access/download;Figure;Fig_1.tiff

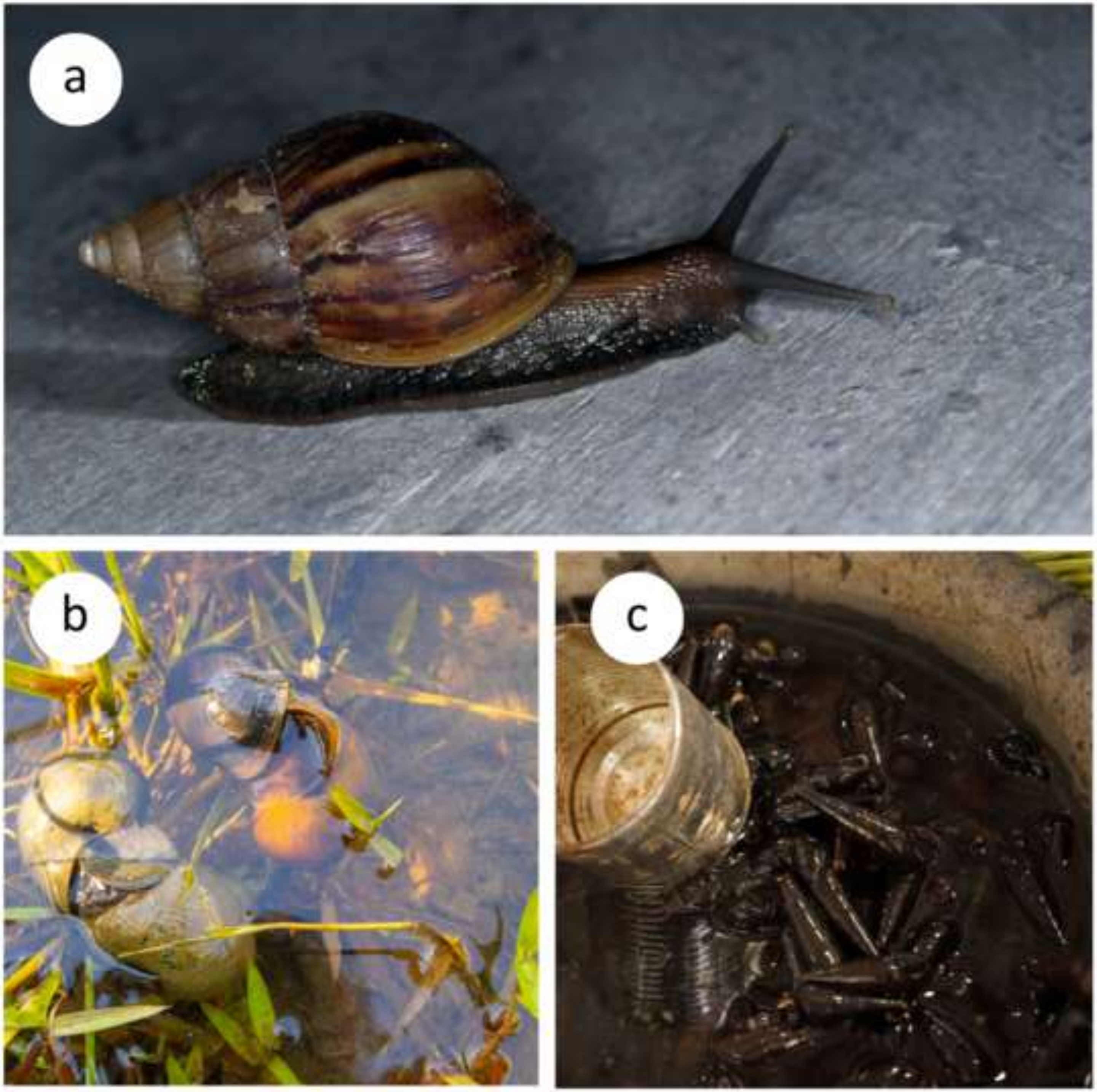

Fig 2

Click here to access/download;Figure;Fig_2.tiff

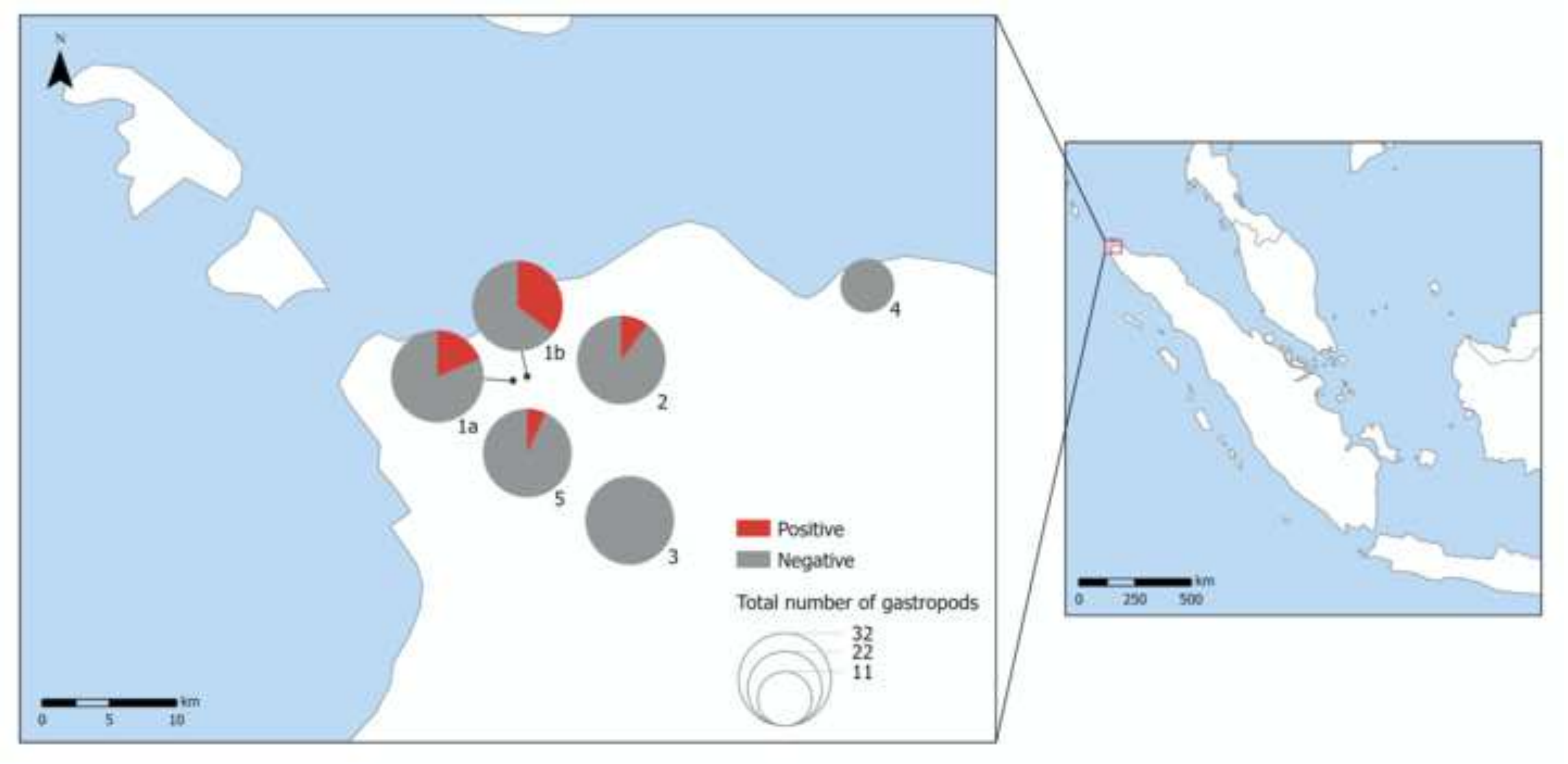

We would like to thank to all the reviewers for their comments and we did our best to make all the suggested changes possible and to reply to each comment individually. Replies to the general comments below.

To the general comments:
The author summary was added.
The authors' contribution section was added.
The trademark symbols were removed.
All the pictures (1, 2 and 3) were obtained by authors during the fieldwork.
The maps were created in ArcGis software with Natural Earth - All maps are public domain.
The role in the study design statement was added to the Financial disclosure statement - section "Funding" at the end of the document.
Conclusion section was added.

Reviewer 1
I suggest that the authors detail the methodology used for each stage of this study, as it can be reproduced by other researchers.
We did our best to rewrite the Methodology section so it is clearer and reproducible

The manuscript is original, it is well written and presents interesting data on the malacological surveillance of natural hosts of A. cantonensis and reinforces Achatina fulica as the intermediate host that most favors the maintenance of the parasite cycle in the environments studied. This has been demonstrated in several countries with a large dispersion of this snail, such as in Brazil. Some other references can be added. I suggest that the authors detail the methodology used for each stage of this study, as it can be reproduced by other researchers. In their results, they could include a comparison between the results of the LAMP and qPCR assays. Both were performed, if I understood correctly. Did any sample present different results between them? Please make this point clearer.
Thank you for the suggestions, we did our best to make both Methods and Results sections of LAMP and qPCR clearer and reproducible. Since LAMP was done on pools only and qPCR on individuals from positive LAMP pools, the suggested comparison is not possible, but we changed the text so it is obvious which assay resulted positive and negative.

In their results, they could include a comparison between the results of the LAMP and qPCR assays. Both were performed, if I understood correctly. Did any sample present different results between them? Please make this point clearer.
We changed the text accordingly.

Pg 1 line 16 - Lissachatina fulica – Lissachatina is a subgenus: Achatina (Lissachatina) fulica. There is not enough phylogenetic support to elevate it to a genus. Many authors have used it as a genus in recent years, which causes a lot of confusion. I suggest changing it throughout the text. The subgenus should be cited only at the beginning of the text. After that, use only "A. fulica"
Corrected in the text
Pg 1 Line 28 – abbreviate A. cantonensis after first mention in the text
Corrected
Pg 1 Line 45 – Please cite examples of freshwater vertebrates

Corrected

Pg 3 Line 61 – Please describe the LAMP assay to help the reader understand better. But only do so in the next topic. How was this pooled analysis performed? What steps were taken? And how much of each mollusk was analyzed?

In the topic "Sample collection (52-76)", I suggest separating the information that actually deals with the collection and, only then, talking about the procedures for parasitological analysis of each group of mollusks. I believe it will be clearer this way.

The next topic deals with molecular analyses. In it, the authors can talk about the LAMP method.

Thank you for the suggestions. We changed the text accordingly and did our best to make the section clearer.

Pg 3, Line 108 – I suggest starting with "The terrestrial snail A. fulica…".

Corrected

Pg 4, Lines 119-121: I suggest: Five DNA sequences of A. cantonensis recovered from A. fulica samples were obtained from two different localities (City Center - park and Nurul Qualbi gardens) ranging from 322 to 554 bp.

Corrected

<u>Reviewer 2</u>

Pls provide the primer sequences of ITS1 with thermal cycle of the ITS1 based PCR

Thank you for the comment, the ITS1 targeted PCR is not originally designed for the purpose of this manuscript, we used the primers and cycling conditions as in Izquierdo-Rodriguez et al., 2023 - as stated in the Methods section. We changed the text so it clearly states which primers were used.

<u>Reviewer 3</u>

- "Notably, the handling and consumption of snails sold at wet markets do not appear to increase the risk of eosinophilic meningitis in this region." May this be because the freshwater snails selled in the wet market do not come from this area? Consider adding some info regarding this point in the discussion. Maybe Sulcospira species from the market are not positive because they come from other areas luckily free of cantonensis. Just an idea, but stating that it do not contribure… well, in this case. Has Sulcospira being reported as IH in other regions? If not, then it might not be a good IH, independently of being selled in the wet market.

Thank you for the suggestion. We changed the text accordingly and explained better in the discussion.

L6: references mention only a few specific cases of the rat lungworm in wildlife. If not reporting all the species reported as accidental, I recomment to reduce the references to the two reviews (ref. 4 and 6). All animal groups mentioned in the rest of references appear in those two reference. In fact, reference 5 is included in the review of the 4…

References were deleted from the text.

L13: Please, put the scientific names of the rat species found in Sumatra in brackets "… also identified in rats (Rattus tiomanicus…. and Rattus…).

Corrected

L15: add here the common name of Lissacatina fulica and delete it from line 19.

We have changed the text so it is clear Line 15 is not referring only to A. fulica (therefore the common name has to remain in Line 19)

L16: Add the S of the plural “South Sumatra ProvinceS”.
Corrected
L19: I would suggest unifying all the snail’s species photographs in a single figure containing the three photos at once. This way it is easier to follow and have a general idea on the species involved in the study.
The images were merged into one figure.
L22: “the abundance of these gastropods in Aceh province is high”. Please, add a reference for this affirmation.
The references were added.
L26: As mentioned above, unify all snail photos in a single figure.
Corrected

I am missing an important part here, the identification of the snails. Although I understand these species may not be difficult to identify, being this study focused on the snails, the correct identification of each genus mentioned is crucial and should be confirmed. Please, add the references used for the identification of the snails. If non used, consider performing some PCR of a few individuals per genus to be sure it is correct. Add a small section with all the methods.
Thank you for the suggestion. In our team, a malacologist determined A. fulica and Pomacea sp., common invasive species - which identification is trivial for an experienced malacologist. In the case of Sulcospira, we added a reference for morphological identification that was used. The reference deals with gastropods in Vietnam, however the studies from Sumatra are missing - and that is why we only determined the Sulcospira specimens only to a genus level.

L62: Why was LAMP used and then qPCR instead of performing just the qPCR? Explain.
We clarified in the text - Methods - LAMP section.
L69: Why was the additional procedure of the emerging of Angiostrongylus performed in these snails instead of analysing just the tissue snips as in Lissachatina? Are we able to compare results using different methods?
We did our best to clarify this in the text - methods as well as below (comment to the Line 143)
L75: Now the Sulcospira (also an aquatic snail) samples are analysed directly without doing the emergence of larvae step. Why is this? Why not performing the same analysis in all snails the same way? Results may differ because of the techniques. Please, clearly explain the reason to differ in the methodology per species.
We did our best to clarify this in the text - methods as well as below (comment to the Line 143)
The preferred method - the one used in Achatina and Sulcospira, could not be performed in Pomacea - see below. But since the aim is to detect DNA of the parasite, it is highly unlikely to miss the DNA if even a larval fragment is present (Sears et al. 2021)
L80: I assume you wanted to say “99ºC”.

Corrected
L85: “with modification optimized for L3 larvae of A. cantonensis, when the pre-lyse phase was extended overnight”. I would rewrite it, as it seems not natural to me using that “when”. Maybe something like “extending the pre-lyse phase overnight as a modification for L3 larvae of A. cantonensis.
I would rewrite the whole Methods section; it is not clear if LAMP was performed in all samples or just in Lissachatina and Pomacea snails. Also, there is different methodology per genus without explanation.
We clarified in the text and changed the text accordingly.

L109: Please, give 95% Confidence Interval (exact method) together with the prevalence (add this in the section methods), and a bit on the descriptive analysis.
We changed the text accordingly.
L104: Please, delete the blank space between 6._7% (6.7%).
Corrected
L117: Fig 3 refers to a photograph of the Sulcospira snails. Do you mean the map of the Fig. 4? Please check. Also, important, ADD scale bar to maps.
The reference was corrected, and a scale bar was added to the map.
L119: I suggest writing “five DNA sequences varying from 322 to 554 bp from …”. In this section I miss a clear result on the LAMP and posterior confirmation of the qPCR. Only final positives are reported, but it would be interesting to know if all LAMP positives were confirmed with qPCR or if there were some false positive pools.
We changed the text accordingly.

L141: “Notably, both A. cantonensis and A. malaysiensis are zoonotic.”. Why this sentence here?
This sentence was removed from the main text
L143: Are we sure on the absence of A. cantonensis in this species as we performed a different methodology?
We applied a different processing method for *Pomacea* sp. compared to other snail species due to anatomical constraints. *Pomacea* snails possess extremely hard shells and a tight-closing operculum, making dissection difficult and potentially hazardous without heavy mechanical force. Crushing them in hard zip-lock bags partially filled with water allowed us to (1) break the shells effectively, (2) pool and process specimens efficiently, and (3) promote larval emergence and sedimentation, as previously described by Modrý et al. (2021). This method is highly sensitive - qPCR assays can detect as little as 1/100,000 of a larva (Sears et al. 2021) - and therefore, we consider it highly unlikely that *A. cantonensis* DNA would have been missed in positive individuals using this approach.
We have changed the text so it is clear why we chose this approach.
L160: I suggest adding these two references together with ref. 19: https://doi.org/10.1371/journal.pone.0094969 and https://doi.org/10.1016/j.onehlt.2023.100610.

The suggested references deal with intermediate hosts of the parasite while the sentence and our provided references are about paratenic hosts.

L166-168: Might this also be driven by rat distribution and densities?

This note was added to the main text

L173: Start the sentence with a “the”. The presence.

Corrected

Maybe a small sentence regarding why you do not differenciate between snail species in this study would be great. Consider adding something similar in the methods’ section.

Clarified in the text.

Ref. 29: Italics in scientific name.

Corrected

Figure 1-3: As mentioned above in the text, it would be clear to have the three snail photos in the same figure. Also, apart from the photo of L. fulica, the rest of the species are not clearly visible. Although these are good pictures of the snails in the environment and in the wet market, consider adding some photos with a clear view of the snail to see the morphology a bit more, especially when no info on the snails’ identification has been provided in the text.

The figures were merged into one. We provided a reference according to which Sulcospira was identified.

Figure 4: Add scale, at least in the zoomed map.

Scale bar was added to the map.

Click here to access/download
Revised Article with Changes Highlighted
Aceh_MS_PLoS 18.7.2025_tracked_changes.pdf
