# Supplementary material for: Hidden threats in urban environments: Angiostrongylus cantonensis in Banda Aceh’s cityscape
Source: PLoS Negl Trop Dis. 2025 Oct 30;19(10):e0013634. doi: 10.1371/journal.pntd.0013634 (PMC12588454; doi:10.1371/journal.pntd.0013634)
Supplement: Attachment — Submitted filename: reviewers answers_3.pdf [file pntd.0013634.s005.pdf]

Reviewer #1: All previous recommendations have been met. The manuscript is better presented.

Reviewer #2: PCR methods are still cryptic. Please provide the detail of the provide the primer sequences of ITS1 with thermal cycle of the ITS1 based PCR. The method must be reproducible. You provided the reference. If any one want to follow your method then he/she need to check the cross ref. Please make the story self-dependent. Otherwise, the manuscript is fine and interesting.

We added the primers and cycling conditions now.

Reviewer #3: Methods section has now improved a lot. I would like to thank the authors for clarifying all my concerns related to the identification of the snails and the methodology used in the analysis. Now is clearly stated why using a different methodology depending on the snails.

I would like to add some minor comments:

- Line 85: I would move the methods regarding to the prevalence calculation and descriptive analysis to the end of the methods section, as we need to know first how do you analyse the snails (LAMP and qPCR methodology), and based on those results, calculate the prevalence.

The paragraph was moved to the the end of Methods section.

- Line 74: You mention here that analysis was performed in pools of 10 individuals for Sulcospira, but no info on pools of the other species was reported here, but it is explained in the LAMP section. As pools were used only for LAMP, I would move all info about pools to the LAMP section, mentioning how many individuals were mixed in pools and why the number differed between species (I suppose it was because of the size of the snail, but it must be mentioned in the text).

We changed the text accordingly.

-Line 106: This is the methods section, so we do not know yet that only A. fulica was positive. Rewrite it in a more general way like: Pools that tested positive to LAMP analysis underwent the species specific qPCR using isolated DNA from each individual included in the pool....

We changed the text accordingly.

Results:

-Does the analysis presented match the analysis plan?

-Are the results clearly and completely presented?

-Are the figures (Tables, Images) of sufficient quality for clarity?

Reviewer #1: All previous recommendations have been met. The manuscript is much better presented.

Reviewer #2: Well done

Reviewer #3: Thank you for answering all my previous comments, now the article gained in clarity and accuracy.

Conclusions:

-Are the conclusions supported by the data presented?

-Are the limitations of analysis clearly described?

-Do the authors discuss how these data can be helpful to advance our understanding of the topic under study?

-Is public health relevance addressed?

Reviewer #1: The findings are relevant and well presented

Reviewer #2: Based on solid proof

Reviewer #3: Yes

Editorial and Data Presentation Modifications?

Use this section for editorial suggestions as well as relatively minor modifications of existing data that would enhance clarity. If the only modifications needed are minor and/or editorial, you may wish to recommend "Minor Revision" or "Accept".

Reviewer #1: I just leave a suggestion that the authors add a reference regarding the identification of Pomacea sp.

The references were added by the malacologists in the team, thank you for the suggestion.

Reviewer #2: (No Response)

Reviewer #3: (No Response)

Summary and General Comments:

Use this section to provide overall comments, discuss strengths/weaknesses of the study, novelty, significance, general execution and scholarship. You may also include additional comments for the author, including concerns about dual publication, research ethics, or publication ethics. If requesting major revision, please articulate the new experiments that are needed.

Reviewer #1: (No Response)

Reviewer #2: (No Response)

Reviewer #3: I think the manuscript has improved a lot and should be accepted for publication after addressing those three minor comments.
