# Supplementary material for: Hidden threats in urban environments: Angiostrongylus cantonensis in Banda Aceh’s cityscape
Source: PLoS Negl Trop Dis. 2025 Oct 30;19(10):e0013634. doi: 10.1371/journal.pntd.0013634 (PMC12588454; doi:10.1371/journal.pntd.0013634)
Supplement: Attachment — Submitted filename: Reviewer.docx [file pntd.0013634.s002.docx]

The manuscript is original, it is well written and presents interesting data on the malacological surveillance of natural hosts of *A. cantonensis* and reinforces *Achatina fulica* as the intermediate host that most favors the maintenance of the parasite cycle in the environments studied. This has been demonstrated in several countries with a large dispersion of this snail, such as in Brazil. Some other references can be added. I suggest that the authors detail the methodology used for each stage of this study, as it can be reproduced by other researchers. In their results, they could include a comparison between the results of the LAMP and qPCR assays. Both were performed, if I understood correctly. Did any sample present different results between them? Please make this point clearer.

Below, I provide some suggestions and other comments will be in the manuscript.

Pg 1 line 16 - *Lissachatina fulica* – *Lissachatina* is a subgenus: *Achatina* (*Lissachatina*) *fulica*. There is not enough phylogenetic support to elevate it to a genus. Many authors have used it as a genus in recent years, which causes a lot of confusion. I suggest changing it throughout the text. The subgenus should be cited only at the beginning of the text. After that, use only “*A. fulica*”

Pg 1 Line 28 – abbreviate *A. cantonensis* after first mention in the text

Pg 1 Line 45 – Please cite examples of freshwater vertebrates

Pg 3 Line 61 – Please describe the LAMP assay to help the reader understand better. But only do so in the next topic. How was this pooled analysis performed? What steps were taken? And how much of each mollusk was analyzed?

In the topic “Sample collection (52-76)”, I suggest separating the information that actually deals with the collection and, only then, talking about the procedures for parasitological analysis of each group of mollusks. I believe it will be clearer this way.

The next topic deals with molecular analyses. In it, the authors can talk about the LAMP method.

Pg 3, Line 108 – I suggest starting with “The terrestrial snail *A. fulica…*”.

Pg 4, Lines 119-121: I suggest: Five DNA sequences of *A. cantonensis* recovered from *A. fulica* samples were obtained from two different localities (City Center - park and Nurul Qualbi gardens) ranging from 322 to 554 bp.
